# Discriminative Metabolomics Analysis and Cytotoxic Evaluation of Flowers, Leaves, and Roots Extracts of *Matthiola longipetala* subsp. *livida*

**DOI:** 10.3390/metabo13080909

**Published:** 2023-08-03

**Authors:** Mona M. Marzouk, Nesrine M. Hegazi, Mona O. A. El Shabrawy, Mai M. Farid, Salwa A. Kawashty, Sameh R. Hussein, Nabiel A. M. Saleh

**Affiliations:** Phytochemistry and Plant Systematics Department, Division of Pharmaceutical Industries, National Research Centre, Cairo P.O. Box 12622, Egypt; nm.hegazi@nrc.sci.eg (N.M.H.); mo.osama@nrc.sci.eg (M.O.A.E.S.); mm.farid@nrc.sci.eg (M.M.F.); sa.kawashty@nrc.sci.eg (S.A.K.); rr.hussein@nrc.sci.eg (S.R.H.); na.saleh@nrc.sci.eg (N.A.M.S.)

**Keywords:** cytotoxicity, HeLa cell line, *Matthiola longipetala*, molecular networking, UPLC-HRMS-MS

## Abstract

*Matthiola longipetala* subsp. *livida* is an annual herb in Brassicaceae that has received little attention despite the family’s high reputation for health benefits, particularly cancer prevention. In this study, UPLC-HRMS-MS analysis was used for mapping the chemical constituents of different plant parts (i.e., flowers, leaves, and roots). Also, spectral similarity networks via the Global Natural Products Social Molecular Networking (GNPS) were employed to visualize their chemical differences and similarities. Additionally, the cytotoxic activity on HCT-116, HeLa, and HepG2 cell lines was evaluated. Throughout the current analysis, 154 compounds were annotated, with the prevalence of phenolic acids, glucosinolates, flavonol glucosides, lipids, peptides, and others. Predictably, secondary metabolites (phenolic acids, flavonoids, and glucosinolates) were predominant in flowers and leaves, while the roots were characterized by primary metabolites (peptides and fatty acids). Four diacetyl derivatives tentatively assigned as *O*-acetyl *O*-malonyl glucoside of quercetin (**103**), kaempferol (**108** and **112**), and isorhamnetin (**114**) were detected for the first time in nature. The flowers and leaves extracts showed significant inhibition of HeLa cell line propagation with LC_50_ values of 18.1 ± 0.42 and 29.6 ± 0.35 µg/mL, respectively, whereas the flowers extract inhibited HCT-116 with LC_50_ 24.8 ± 0.45 µg/mL, compared to those of Doxorubicin (26.1 ± 0.27 and 37.6 ± 0.21 µg/mL), respectively. In conclusion, the flowers of *M. longipetala* are responsible for the abundance of bioactive compounds with cytotoxic properties.

## 1. Introduction

Brassicaceae (=Cruciferae) is one of the economically important angiosperm families, commonly known as the crucifers, cabbage, or mustard family, containing over 372 genera and approximately 4636 species [1]. Plants of the family Brassicaceae have been an interesting research subject for years due to their economic and agricultural importance. Many species have been valued as food crops; some are vegetables, others are sources of industrial and cooking oils, forage, and condiments and others are grown as ornamental species for their showy flowers and significant numbers as medicinal herbs [2]. Additionally, certain wild cruciferous plants are rich in secondary metabolites, especially glucosinolates, phenolic acids, and flavonoids, which have many biological activities and, therefore, numerous nutritional and medicinal benefits [3,4]. *Matthiola longipetala* subsp. *livida* (Delile) Maire is one of the common wild medicinal cruciferous herbs growing mainly in the Egyptian Mediterranean region, and it is locally known as “Manthor” [5]. Although some previous phytochemical studies have been conducted on *M. longipetala* subsp. *livida* [6,7,8,9], the reported compounds represent only a small portion of the species’ chemical composition. Similarly, certain biological activities such as antibacterial, antifungal, and anticancer effects have been reported for the investigated species [8,9,10].

Lately, metabolomics platforms have been widely used to map the metabolome of plants, among which ultra-performance liquid chromatography coupled with high-resolution tandem mass spectrometry (UPLC-HRMS/MS) as the most extensively adopted for mapping the secondary metabolome space. UPLC-HRMS/MS offers the advantages of high efficiency, reproducibility, and shorter analysis [11]. Additionally, advances in the data analysis tools, such as molecular networks through the Global Natural Products Social Molecular Networking (GNPS) [12], allow for the visual display of the constitutive metabolome among samples and the propagation of metabolites annotation [13].

Over the last few decades, most new therapeutic interventions involving plant secondary metabolites and their derivatives have been aimed at combating cancer. In this regard, cruciferous plants have been previously reported to lower the risk of developing various cancers [14]. Our previous research reported the moderate cytotoxic potential of the alcoholic extract of the aerial part of *M. longipetala* subsp. *livida* against cervix (HeLa) and colon (HCT116) cell lines [8]. Moreover, another report assessed the extract’s activity against HepG2 cells in vitro using MTT, DNA fragmentation, and cell proliferation cycle measurements, and it demonstrated significant activity [15].

In continuation of our previous study, the present work aimed to map the under-explored chemistries of different organs (i.e., flowers, leaves, and roots) of *M. longipetala* using UPLC–HRMS-MS analysis that recruited for a holistic overview of the plant’s constitutive chemistries, coupled with spectral similarity networks through the GNPS [12]; this was in addition to evaluating the cytotoxic activities of the three organs on HCT-116, HeLa, and HepG2 cell lines to suggest the one responsible for this potential.

## 2. Materials and Methods

### 2.1. Chemicals and Reagents

All chemicals for chemical analysis were obtained from Sigma-Aldrich (Merck, Kenilworth, NJ, USA).

### 2.2. Plant Material and Preparation of the Extracts

*M. longipetala* subsp. *livida* (650 g fresh weight) was collected from Alexandria-Marsa Matruh Road, 31°04′15.3″ N 27°58′10.4″ E, Egypt, in February 2018. The identity of the plant was authenticated by Prof. Dr. Mona M. Marzouk. A voucher specimen (ML_28_2_18) was placed in the herbarium of the National Research Centre (CAIRC), Cairo, Egypt. The flowers, leaves, and roots (117, 175, and 152 g fresh weight, respectively) were washed thoroughly with bi-distilled H_2_O, dried in shade, and ground finely. Fifteen grams of each dried powdered organ was separately extracted using 70% methanol (500 mL) by sonication (2 h, 60 °C) and filtered over charcoal to yield three aqueous methanolic extracts [16,17]. The flowers, leaves, and roots extracts were concentrated under reduced pressure at 50 °C to produce three dried extracts (2.761, 1.140, and 1.832 g, respectively).

### 2.3. Sample Preparation for UPLC-HRMS-MS Measurement

The dried extracts were prepared for UPLC-HRMS/MS analyses following a previously described protocol [16]. The extracts (50 mg each) were dissolved in 70% MeOH (HPLC-grade) with sonication (10 min), then centrifuged. Aliquots were then evaporated under reduced pressure, followed by freeze-drying for 48 h. For MS analysis, 1 mg in 250 µL MeOH (MS-grade) were prepared consuming 5 µL as an injection volume in the UPLC-MS analysis.

### 2.4. UPLC-HRMS-MS Analysis

The HRMS/MS analysis was conducted on a MaXis 4G instrument (Bruker Daltonics^®^, Bremen, Germany) coupled with an Ultimate 3000 UPLC (Thermo Fisher Scientific^®^, Waltham, MA, USA). A UPLC method was applied as described by [17] as follows: (with 0.1% formic acid in H_2_O as solvent A and 100% ACN as solvent B), an isocratic gradient of 10% B for 10 min, 10% to 100% B in 30 min, 100% B for an additional 10 min, using a flow rate of 0.3 mL/min; 5 µL injection volume and UV detector (UV/VIS) wavelength monitoring at 210, 254, 280, and 360 nm. The separation was conducted on a Nucleoshell RP 18 column, 2.7 µm, 150 × 2 mm (Macherey-Nagel^®^, Düren, Germany), and the range for MS acquisition was 50–1800 Daltons (Da). A capillary voltage of 4500 V, nebulizer gas pressure (nitrogen) of 2 (1.6) bar, ion source temperature of 200 °C, dry gas flow of 9 L/min, and spectral rates of 3 Hz for MS^1^ and 10 Hz for MS^2^, were utilized. For acquiring MS/MS fragmentation, the 10 most intense ions per MS^1^ were selected for subsequent CID, with stepped CID energy applied. The employed parameters for tandem MS were applied as previously detailed [18].

### 2.5. Data Analysis and Preprocessing

Raw data inspection was performed using Compass Data Analysis 4.4 (Bruker Daltonics^®^). A Metaboscape 3.0 (Bruker Daltonics^®^) was utilized for feature detection, grouping, and alignment, employing the T-ReX 3D (Time aligned Region Complete eXtraction) algorithm [19]. Bucketing was performed with an intensity threshold of 1 × 10^5^ and a retention time range from 0 to 40 min with a restricted mass range *m*/*z* from 130 to 1800.

### 2.6. Feature-Based Molecular Networking (FBMN) and Metabolites Dereplication

The produced MGF file and the feature quantification table (CSV file) were used in the feature-based molecular networking (FBMN) following the online workflow in GNPS platform (http://gnps.ucsd.edu), accessed on 28 December 2019 [20]. The parameters applied for the construction of the FBMN via the GNPS platform as follows: a parent mass tolerance (0.05 Da), a fragment ion tolerance (0.05 Da), a cosine score (0.7), and minimum shared fragments (6). To avoid misinterpretation of artifacts, the blank run was uploaded as a distinct sample on GNPS workflow and excluded from the networks. Cytoscape version 3.9.1 (https://cytoscape.org/), accessed on 28 February 2022, was used for the network visualization.

The metabolites’ dereplication was based on the chromatographic performance, chemical formula, and fragmentation pattern compared to those of MS^2^ data from literature and spectra from MS reference database (MoNA, NIST14, and Respect) (Table 1). Sirius plus CSI:FingerID 5.5.4 were used for the manual putative structures identification [21], assisted by the molecular formula prediction (C, H, N, O, S, and P) and candidate search with *m*/*z* tolerance set to 20 ppm connected to online Pubchem. The proposed in silico fragmentation trees are the impetus for further support for identification.

### 2.7. Cytotoxic Activity

#### 2.7.1. Cell Lines

Human tumor cell lines: the colon carcinoma (HCT116), cervix carcinoma (HeLa), and hepatocellular carcinoma (HepG2) cell lines were supplied by Vacsera (Giza, Egypt) and maintained at the Bioassay-Cell Culture Laboratory, National Research Centre, El-Bohouth St., Dokki, Cairo 12622, Egypt.

#### 2.7.2. Cell Viability by MTT Assay

The samples were prepared by dissolving stock solution in DMSO to give operating concentrations of each sample range from 100 to 0.78 µg/mL, and the cells were incubated with these concentrations in triplicate (37 °C, 72 h) in a CO_2_ environment. Control wells were treated with the same amount of complete growth media only. For all treatments and untreated control groups, complete growth media without cells were added as a blank to reduce the background absorbance values. Separately, each experiment were conducted three times. MTT assay was performed by removing the medium quietly and adding MTT solution (10 μL) with a last concentration (5 mg/mL) per well then incubating (37 °C, 4 h) until the purple crystals were shaped. Then, the MTT solution was discarded from every well and DMSO (100 μL) was subjected to dissolve the crystals. The 96-well plate was shaken (15 min) using a microplate shaker until totally dissolved of the crystals. For each well, the absorbance value was assayed (595 nm wavelength) using a microplate multi-well reader [63]. The cell viability (CV) percentage after treatment with *M. longipetala* subsp. *livida* extracts were considered as follows: CV (%) = (absorbance of the treated cells − absorbance of blanks)/(absorbance of control cell − absorbance of blanks) × 100. The lethal concentration of the samples caused the death of 50% of cells (LC_50_) which was also calculated at 48 h. Doxorubicin, the anticancer drug, was used as a positive control.

## 3. Results and Discussion

### 3.1. UPLC-HRMS/MS Metabolites Profiling of the Extracts

The current study aimed to comparably chart the metabolic composition of different organs (i.e., flowers, leaves, and roots) of *M. longipetala* via UPLC-PDA-ESI-HRMS/MS analysis in both positive and negative ionization modes. The overlaid BPC (base peak chromatograms) of the three extracts exhibited some differences, especially at the Rt range of (10–25 min) in the positive ionization mode and (6–15 min) in the negative ionization mode (Supplementary Figure S1), suggesting that the three extracts could be of different biological relevance.

### 3.2. UPLC-HRMS/MS Metabolite Annotation Aided with Molecular Networking

The UPLC-HRMS/MS data were mined employing the GNPS platform, in which feature-based molecular networks (FBMNs) were generated to visually display the existing chemical space and the metabolites distribution in the different plant parts of *M. longipetala*.

**Figure 1:**
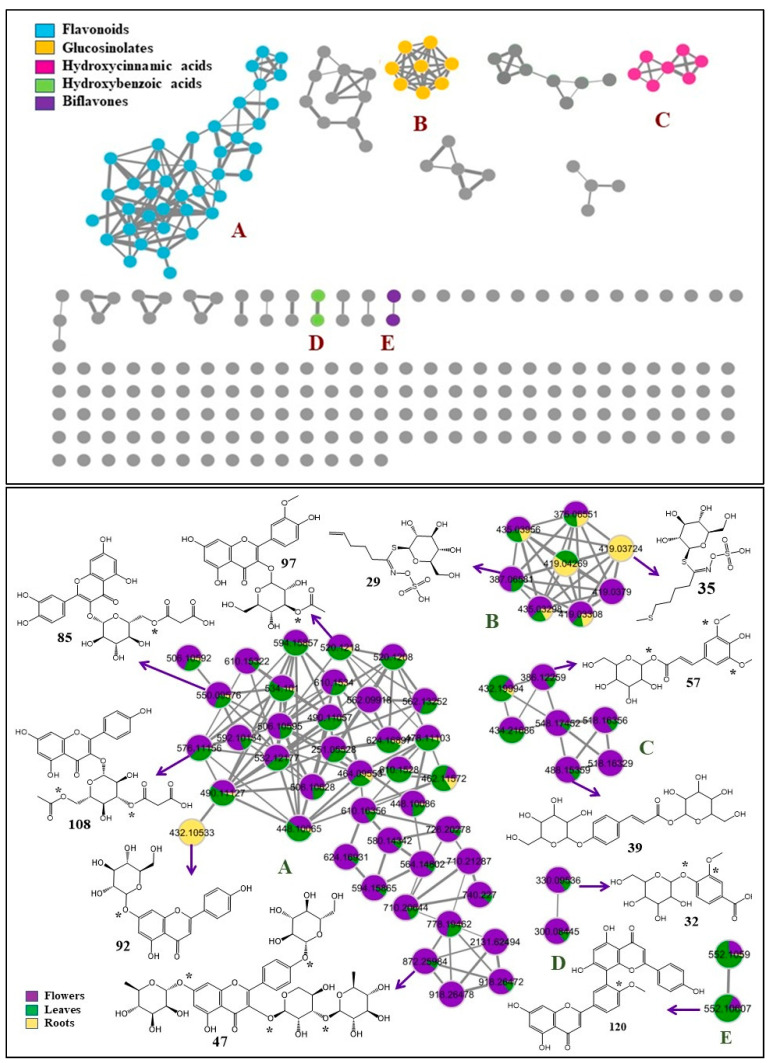
FBMN created using MS/MS data of *Matthiola longipetala* subsp. *livida* extracts (negative ionization mode): flowers (purple), leaves (green), and roots (yellow) extracts. Cluster A (flavonoid glucosides), Cluster B (glucosinolates), Cluster C (hydroxycinnamic acids), Cluster D (hydroxybenzoic acids), and Cluster E (biflavones). *; the substitution position may vary.

**Figure 2:**
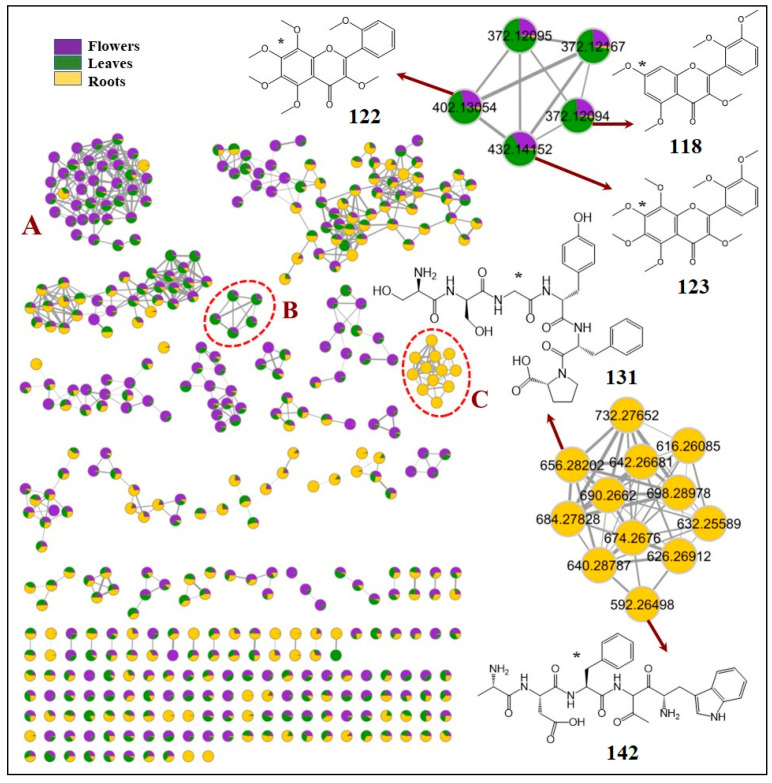
FBMN created using MS/MS data of *Matthiola longipetala* subsp. *livida* extracts (positive ionization mode): flowers (purple), leaves (green), and roots (yellow) extracts. Cluster A (flavonoid glycosides and hydroxylated flavonoid aglycones), Cluster B (methoxylated flavonoid aglycones), and Cluster C (peptides). *; the substitution position may vary. The red circles explained the enlarged clusters.

Two FBMNs were laid out from the acquired MS/MS data for both ionization modes. The negative FBMN constituted 188 nodes grouped into 19 clusters (with a minimum of two connected nodes) and 130 singletons. The significant dereplicated sets of the negative FBMN were the secondary metabolites clusters: A (flavonoid glycosides), B (glucosinolates), C (hydroxycinnamic acid derivatives), D (hydroxybenzoic acid derivatives), and E (biflavones) (Figure 1). These metabolites are distributed in the flowers and leaves organs with their abundance in flowers, which could be responsible for the current cytotoxic assessment and guidance for further biological activities. Similarly, the positive FBMN constituted 257 nodes in 41 clusters and 104 discrete nodes, in which the classes of interest are cluster A (flavonoid glycosides and hydroxylated flavonoid aglycones) and B (methoxylated flavonoid aglycones); besides, cluster C (peptides) is presented as a primary metabolites class which characterized the roots organ and ionized in the positive ionization mode only (Figure 2). In general, nodes were portrayed as a pie chart to reflect the relative abundance of each ion in the three plant parts.

In total, 154 compounds were annotated belonging to different chemical classes (i.e., glucosinolates, phenolic acids, flavonoids, etc.). Almost all the annotated features are reported for the first time to exist in *M. longipetala* subsp. *livida* (Table 1). The classes and/or subclasses of compounds were preformed manually guided by the literature [2,28,33,80,81] and automatically through the ClassyFire webserver at http://classyfire.wishartlab.com/ (accessed on 27 June 2023) [82]. Following is a detailed discussion of the detected metabolites according to their chemical class.

#### 3.2.1. Glucosinolates

Glucosinolates are one of the main bioactive metabolites of the Brassicaceae species and are thought to play a significant role in the health benefits of such species [2,28,80]. Their fragmentation behavior involves the cleavage of the sugar–sulfur bond, giving the fragment ion *m*/*z* 259 and the sulfur-aglycone showing fragment ions at *m*/*z* 195 and *m*/*z* 275. The intramolecular rearrangements of the attachment of aglycone and sulfate to the glucose moiety give the fragment ion *m*/*z* 241 after water cleavage from *m*/*z* 259 [28,80].

Eight of the nine identified glucosinolates are grouped in cluster B of the negative FBMN, occurring in the three plant parts (Figure 1). This includes isomers of glucoraphanin (**7** and **14**, *m*/*z* 434.0253 [M − H]^−^), together with isomers of methylthio-butenyl-glucosinolate (**11**, **18**, **23** and **35**, *m*/*z* 418.0299 [M − H]^−^), butyl glucosinolate (**20**, *m*/*z* 374.0582 [M − H]^−^), and glucobrassicanapin (**29**, *m*/*z* 386.0582 [M − H]^−^). Lastly, one glucosinolate was observed in the positive FBMN as a self-looped node and was identified as raphenin (**10**, *m*/*z* 176.0201 [M − H]^+^) (Table 1).

#### 3.2.2. Phenolics

Besides the glucosinolates, members of the Brassicaceae are well recognized for their high content of phenolic metabolites, with qualitative and quantitative differences among species and varieties, within the same species, and plant parts [33]. In the present study, phenolic metabolites showed the highest accumulation in the flowers extract and the least in the roots. The major phenolic classes identified were phenolic acids and flavonoids.

##### Phenolic Acids and Derivatives

Detected phenolic acids included hydroxybenzoic acid and hydroxycinnamic acid (coumaric, ferulic, and sinapic acids) derivatives, which are widely distributed in numerous members of the Brassicaceae family, commonly as glycosylated descendants [2,33].

The negative FBMN delineated the abundance of glycosylated hydroxycinnamic acids in the flowers and grouped in cluster C (Figure 1), including isomers of coumaric acid-*O*-dihexoside (**12** and **39**, *m*/*z* 487.145 [M − H]^−^), isomers of ferulic acid-*O*-dihexoside (**26** and **43**, *m*/*z* 517.155 [M − H]^−^), sinapic acid-*O*-dihexoside (**46**, *m*/*z* 547.167 [M − H]^−^), and later sinapic acid-*O*-hexoside (**57**, *m*/*z* 385.1141 [M − H]^−^). Caffeic acid (**93**, *m*/*z* 179.0354 [M − H]^−^) was also observed in the negative FBMN, but as a self-looped node and also accumulated in the flowers organ.

Similarly, glycosylated hydroxybenzoic acids were distributed in the three organs, and were observed in the negative FBMN (Figure 1). Hydroxybenzoic acid-*O*-hexoside (**19**; *m*/*z* 299.0770 [M − H]^−^), and vanillic acid-*O*-hexoside (**32**; *m*/*z* 329.0853 [M − H]^−^) were viewed as a cluster of two connected nodes (D). Other glycosides were observed as self-looped nodes and identified as dihydroxybenzoic acid-*O*-hexoside (**25**; *m*/*z* 315.0716 [M − H]^−^) and dihydroxybenzoic acid-*O*-pentoside (**37**; *m*/*z* 285.0614). Lastly was dihydroxybenzoic acid (**75**; *m*/*z* 153.0192 [M − H]^−^) which existed only in the roots extract. Additionally, one sulfo-hydroxybenzoic acid (**36**; *m*/*z* 246.9917 [M − H]^−^) was noted as a self-looped node in negative FBMN, showing the characteristic loss of a sulfate group (−80 Da) and was assigned as vanillic acid-sulfate.

Other phenolic derivatives were also observed as self-looped nodes either in the positive or negative FBMN. They also distributed in the three extracts with richness in the flowers and tentatively identified as mono-hydroxy benzaldehyde isomers (**54** and **61**; *m*/*z* 137.0601 [M + H]^+^), trimethoxy benzaldehyde (**69**; *m*/*z* 197.0813 [M + H]^+^), trimethoxy benzaldehyde-*O*-hexoside (**67**; *m*/*z* 357.1559 [M − H]^−^), sinapaldehyde (**84**; *m*/*z* 207.0665 [M − H]^−^), coniferin (**99**; *m*/*z* 341.1242 [M − H]^−^), and syringin (**101**; *m*/*z* 371.1350 [M − H]^−^).

##### Flavonoids

Flavonoids protect plants from various biotic and abiotic stresses by acting as natural antioxidants, unique UV filters, signal molecules, allelopathic compounds, and antimicrobial defensive compounds [81]. Additionally, their impressive biological effects have made them excellent candidates as nutraceutical supplements for human intake, disease prevention, and health promotion [2,81].

Throughout the current analysis, around 40% of the detected constituents are flavonoids (64 metabolites) (Table 1) delivered as cluster A and some as self-looped nodes in FBMN of the negative ionization mode (Figure 1) and clusters A and B in the positive one (Figure 2), being more abundant in the flowers.

Flavonoid-*O*-glycosides

The flavonoid-*O*-glycosides (56 compounds) were represented in cluster **A** in both FBMNs (Figure 1 and Figure 2), mainly as flavonol-*O*-glycosides, which have been previously reported in various species of Brassicaceae [2,3,4,28,31,39,45,46,48,65,83]. They were mostly distributed among the three investigated plant organs, with more abundance in the flowers. Only one flavone-*O*-glycoside was detected exclusively in the roots and was assigned as apigenin 7-*O*-glucoside (**92**, *m*/*z* 431.0981 [M − H]^−^) based on the main fragment ion at *m*/*z* 269 which corresponds to apigenin aglycone and the loss of a glucose moiety [M − H–162]^−^.

Flavonol-*O*-glycosides

The predominant annotated flavonol glycosides were mainly glycosides of kaempferol, isorhamnetin, and quercetin with little presence of rhamnocitrin, based on our former studies through acid hydrolysis and NMR data [8,46]. The quercetin glycosides in both FBMN were directly linked to their kaempferol correspondences by a difference of 16 Da (–O–), and with the isorhamnetin correspondences by a difference of 14 Da (–CH_2_). The direct attachment of the isorhamnetin glycosides to those of the kaempferol correspondents with a mass difference of 30 Da suggests possible OCH_3_ expansion.

The flavonol-O-glycosides showed the typical fragmentation patterns corresponding to the respective sugar moiety, such as deoxyhexose (−146 Da), hexose (−162 Da), and pentose (−132 Da). Mostly, the sugar moieties were tentatively assigned as rhamnose, glucose, and arabinose based on previous studies with acid hydrolysis and NMR data of the investigated species [8,46] and several members of the family Brassicaceae [3,28,46,65,83]. Some glycosides were acylated by acetic acid (−42 Da) and/or malonic acid (−86 Da).

Twelve flavonol mono-glycosides were observed and reported previously in some crucifers [46,83]. The 3-*O*-glucoside of quercetin (**81**, *m*/*z* 463.0878 [M − H]^−^), kaempferol (**88**, *m*/*z* 447.0931 [M − H]^−^), isorhamnetin (**90**, *m*/*z* 477.1035 [M − H]^−^), and rhamnocitrin (**111**, *m*/*z* 461.1085 [M − H]^−^), the 3-*O*-rhamnoside of kaempferol (**102**, *m*/*z* 431.0980 [M − H]^−^), as well as the 3-*O*-arabinoside of quercetin (**89**, *m*/*z* 433.0755 [M − H]^−^), which were tentatively identified according to Ablajan et al. [49], and Qin et al. [47]. In this case (3-*O*-glycosides), the intensity of the anion radical fragment [Agl–H–H]^−^ is higher than the anion one [Agl–H]^−^ and *vis versus* for **65** (kaempferol 7-*O*-rhamnoside) and **74** (isorhamnetin 7-*O*-rhamnoside).

Additionally, 12 flavonol di-*O*-glycoside structures were annotated and were grouped within the same cluster (Figure 1, cluster A). Compounds (**40**, **60**, and **73**) were assigned as kaempferol di-*O*-glycosides, showing the same molecular ion peak at *m*/*z* 609 [M − H]^−^ and common MS fragments at *m*/*z* 447 and 285. The MS fragmentation pattern of compound **40** is typical for kaempferol 3-*O*-sophroside. This compound revealed the deprotonated base peak at *m*/*z* 285 [M − H–324]^−^, a fragment ion at 429 [M − H–180]^−^, and a fragment ion at 447 [M − H–162]^−^, suggesting a sophoroside at the 3-*O* position [39]. Conversely, the absence of the fragment ion [M – H–180]^−^ in compound **73**, indicates a kaempferol 3-*O*-gentobioside structure [39,49]. In contrast, the appearance of the fragment ion *m*/*z* 447 as the base peak confirmed the identification of compound **60** as kaempferol-3,7-di-*O*-glucoside [49]. Additionally, two *O*-rutinoside isomers of kaempferol (**48**, *m*/*z* 593.1511 [M − H]^−^, **82**, *m*/*z* 593.1580 [M − H]^−^, 595.1665 [M + H]^+^) were confirmed. Sophoroside and rutinoside substitution have been observed in several cruciferous species as predominant disaccharide moieties [39].

Flavonol di-*O*-glycosides with sugar units at different hydroxyl positions of the aglycone nucleus provide two flavonol monoglycoside fragment ions with different intensities, where the higher fragment represents the 3-*O*-substitution while the lower one indicates the occupation of position 7 [39,47,49]. Consequently, compounds **62**, **72**, and **76** could be identified as 3-*O*-rhamnoside 7-*O*-arabinoside of quercetin (*m*/*z* 579.1349 [M − H]^−^), kaempferol (*m*/*z* 563.1398 [M − H]^−^), and isorhamnetin (*m*/*z* 593.1509 [M − H]^−^), respectively. Likewise, the MS fragmentation of compounds **59** and **71** is typical for the 3-*O*-rhamnoside 7-*O*-glucoside of quercetin (*m*/*z* 609.1464 [M − H]^−^) and isorhamnetin (*m*/*z* 623.1633 [M − H]^−^), respectively.

Furthermore, different triglycosides of kaempferol (**27**, **42**, **49**, **50**, **55**, **64**, **66,** and **68**), quercetin (**51** and **58**), and isorhamnetin (**53**) were also grouped in cluster A of both FBMNs (Figure 1 and Figure 2). The MS fragmentation of compound **27** (*m*/*z* 773.2147 [M + H]^+^) was characteristic of kaempferol-3-*O*-sophroside-7-*O*-glucoside [39]. Four kaempferol triglycosides isomers (**50**, **55**, **64**, and **66**) showed a common molecular formula (C_32_H_38_O_18_), molecular ion peaks (*m*/*z* 709 [M − H]^−-^ and 711 [M + H]^+^), and MS fragments at *m*/*z* (431 [M − H–296]^−^ and 433 [M + H–296]^+^), after the neural loss of a disaccharide residue (rhamnosyl arabinoside) and (285 [Agl − H]^−^, 287 [Agl + H]^+^). These compounds were directly connected with compound **72** (*m*/*z* 563.1398) in the negative MN with a mass difference 146 Da (rhamnosyl) (Figure 1), therefore, they could be annotated as kaempferol *O*-rhamnosyl arabinoside-*O*-rhamnoside isomers, one of them is recommended to be kaempferol 3-*O*-(2″-α-rhamnopyranosyl)-β-arabinopyranoside-7-*O-*α-rhamnopyranoside which was isolated before from the investigated plant by Marzouk et al. [8]. Likewise, compound (**58**, *m*/*z* 725.1937 [M − H]^−^ and 727.2094 [M + H]^+^) was linked with compound **62** (*m*/*z* 579. 1349 [M − H]^−^ and 581.1510 [M + H]^+^) in both FBMNs and could be identified as quercetin *O*-rhamnosyl arabinoside-*O*-rhamnoside. Also, the *O*-rhamnosyl arabinoside-*O*-glucoside derivatives of kaempferol (**49**, *m*/*z* 727.2088 [M + H]^+^), quercetin (**51**, *m*/*z* 741.1881 [M − H]^−^ and 743.2038 [M + H]^+^), and isorhamnetin (**53**, *m*/*z* 757.2199 [M + H]^+^) were identified. Based on previous studies, three kaempferol triglycosides **42** (*m*/*z* 757.2199 [M + H]^+^), **52** and **68** (*m*/*z* 739.2087 [M − H]^−^, 741.2243 [M + H]^+^) were confirmed as kaempferol-*O*-glucoside-*O*-rutinoside, kaempferol-*O*-rhamnoside-*O*-rutinoside, and kaempferol-*O*-rhamnosyl rutinoside, respectively [46,51].

Lastly, the highest glycosylation pattern was found in two tetra glycosides of kaempferol (**33** and **47**) which are concentrated in the flowers extract. Compound (**33**) appeared at *m*/*z* 917.2648 [M + FA − H]^−^, while **47** appeared at *m*/*z* 871.2510 [M − H]^−^, they have the same molecular formula (C_38_H_48_O_23_) and MS fragments (*m*/*z* 709 [M − H − 162 (glucose)]−, 563 [Agl − H + 278 (arabinosyl rhamnoside)]^−^, 447 [Agl − H + 162 (glucoside)]^−^, 431 [Agl − H + 146 (rhamnoside)]^−^, 285 [Agl − H]^−^). Thus, they tentatively identified as kaempferol-*O*-arabinosyl-rhamnoside-*O*-rhamnoside-O-glucoside isomers, one of them could be identified as kaempferol 3-*O*-(2″-rhamnopyranosyl)-arabinopyranoside-7-*O*-rhamnopyranoside-4′-*O*-glucopyranoside which was isolated before from the current species [8].

Acylated flavonol-*O*-glycosides

A total of 16 acylated flavonol mono-glycosides were also observed in group A of the positive and negative FBMNs and connected with their *O*-glucoside analogs with MS differences of either 42 Da (acetyl) and/or 86 Da (malonyl). Whereas the acetylated and malonylated counterparts were correlated with each other with a 44 Da (CO_2_) difference (Figure 1 and Figure 2). The 3-*O*-malonyl glucoside of quercetin (**85**, *m*/*z* 549.0881 [M − H]^−^), kaempferol (**95**, *m*/*z* 533.0936 [M − H]^−^), isorhamnetin (**96**, *m*/*z* 565.1198 [M + H]^+^), and rhamnocitrin (**113**, *m*/*z* 549.1246 [M + H]^+^) were characterized by the neutral loss −86 Da (malonyl), then −162 Da (glucoside). Other mono-acylated flavonol glycosides were annotated as kaempferol 3-*O*-acetyl glucoside (**105**, *m*/*z* 489.1035 [M − H]^−^*, m*/*z* 491.1192 [M + H]^+^), two isomers of quercetin 3-*O*-acetyl glucoside (**86** and **94**, *m*/*z* 505.0979 [M − H]^−^*, m*/*z* 507.1142 [M + H]^+^), and three isomers of isorhamnetin 3-*O*-acetyl glucoside (**97, 104**, and **107**, *m*/*z* 519.1143 [M − H]^−^, *m*/*z* 521.129 [M + H]^+^) (Supplementary Figure S2). Acylated monoglycoside derivatives of quercetin, kaempferol, and isorhamnetin have already been found in some cruciferous species [3,39], while reported for the first time from the genus *Matthiola*.

Similarly, the diacylated flavonol glycosides were represented as 3-*O*-diacetyl glucoside of kaempferol (**109**, *m*/*z* 531.1138 [M − H]^−^), and isorhamnetin (**110** and **115**, *m*/*z* 561.12 [M − H]^−^) (Supplementary Figure S2), connected with their 3-*O*-glucoside analogs with MS difference of 84 Da (2 acetyl residues) in negative FBMN (Figure 1). Four additional diacylated flavonol glycosides were 3-*O*-acetyl malonyl glucoside of quercetin (**103**, *m*/*z* 591.0995 [M − H]^−^, *m*/*z* 593.1148 [M + H]^+^), kaempferol (**108** and **112**, *m*/*z* 575.1044 [M − H]^−^*, m*/*z* 577.1201 [M + H]^+^), and isorhamnetin (**114**, *m*/*z* 607.1295 [M + H]^+^). They were linked with their 3-*O*-acetyl glucoside or 3-*O*-malonyl glucoside derivatives with a difference of 86 Da (malonyl) or 42 Da (acetyl), respectively, in either the negative or positive FBMNs (Figure 1 and Figure 2). For instance, compound **103** showed a deprotonated molecular ion peak at *m*/*z* 591.0995 [M − H]^−^ and produced fragment ions at *m*/*z* 547 [M − H − 44]^−^ after the neutral loss of CO_2_ then *m*/*z* 505 [M − H − 86]^−^, for malonyl elimination then *m*/*z* 301 [M − H − 86 − 42]^−^ and *m*/*z* 300 [M − H − H − 86 − 42]^−^, after the loss of the acetyl group (Supplementary Figure S3). Therefore, compound **103** could be identified as quercetin 3-*O*-X_1_ acetyl X_2_ malonyl glucoside. Similarly, compounds (**108** and **112**) were identified as kaempferol 3-*O*-X_1_ acetyl X_2_ malonyl glucoside and **114** as isorhamnetin 3-*O*-X_1_ acetyl X_2_ malonyl glucoside (Supplementary Figures S4 and S5). These four structural proposals were not found before in nature.

Flavonoid aglycones

Five polymethoxylated flavone-type aglycones were mainly observed in the positive ionization mode and represented as a cluster (B) of the FBMN (Figure 2). On the bases of GNPS libraries, they could be annotated as tangeretin (**118**), sinensetin (**119**), and 3,5,7,3′,4′pentatamethylflavone (**124**), all at *m*/*z* 373 [M + H]^+^, irigenin trimethyl ether (**122**) at *m*/*z* 403.1393 [M + H]^+^, and 3,5,6,7,3′,4′,5′heptamethylflavone (**123**). The polymethoxylated flavone aglycones were reported before from some species of the family Brassicaceae [27,33]. Likewise, one flavonol-type aglycone was linked in cluster (A) of the positive FBMN (Figure 2) and annotated as kaempferol (**87**, *m*/*z* 287.0505 [M + H]^+^) that was a predominant structure for all family members [39,46,47,83].

Additionally, two biflavone-structure were detected as a cluster (E) in a negative FBMN and elucidated as two isomers of methylamentoflavone (**120** and **121**, at *m*/*z* 551.09 [M − H]^−^), confirmed by their fragmentation pattern and GNPS library (Figure 1). Rare biflavone derivatives were reported before for some species of Brassicaceae [48].

#### 3.2.3. Iridoids and Diterpenes

Only one iridoid compound was found for the first time in the investigated species and concentrated in the flower parts. The iridoid is identified as loganic acid (**30**) and has a molecular ion peak *m*/*z* 375.1297 [M − H]^−^ and fragment ions at *m*/*z* 213 [M − H − 162]^−^. Similarly, one diterpene structure was identified as miltirone (**127**, *m*/*z* 283.1698 [M − H]^+^) and produced fragment ions at *m*/*z* 265 ([M + H − H_2_O]^+^) and *m*/*z* 223 ([M + H − H_2_O − C_3_H_6_]^+^). Both compounds showed a wide range of activities including anti-cancer, anti-inflammatory, and antioxidant effects [36,71].

#### 3.2.4. Coumarin

Coumarins are another vital class of secondary metabolites and were mainly observed in the positive ionization mode (Table 1). Hydroxy coumarin (**38**, *m*/*z* 163.0605 [M + H]^+^) revealed ions at *m*/*z* 147 [M + H–O]^+^, and 119 [M + H–CO_2_]^+^. Two isomers of dimethoxycoumarin (**45**, **56**, *m*/*z* 207.065 [M + H]^+^) exhibited two characteristic fragments at *m*/*z* 193 [M + H−CH_2_]^+^, and 179 [M + H−2CH_2_]^+^, after loss of 14 Da. Additionally, compound **79** (*m*/*z* 455.1164 [M + H]^+^) was directly connected to **45** and **56**, with a mass difference (248 Da). It produced fragment ions at 411, 369, and 207 after the loss of 42, 44, and then 162 Da, respectively, suggesting the presence of -*O*-malonyl glucoside dimethoxy coumarin.

#### 3.2.5. Amino Acids, Organic Acids, and Derivatives

The annotation of the amino acids was derived from the abundant fragments of the protonated ions and their corresponding derivatives arising from either losing H_2_O (−18 Da) yielding their residue mass or the loss of (H_2_O + CO) (−46 Da) producing their immonium ions [84] leading to the detection of five amino acids including arginine (**1**), proline (**2**), leucine/isoleucine (**17**), phenylalanine (**22**), tryptophan (**34**), and three amino acids derivatives; methyl proline (**5**), dimethyl proline (**6**) and tryptophan *N*-glucoside (**31**), mainly distributed among the three plant organs. Similarly, five organic acids were detected as self-looped nodes either in the negative or positive FBMNs and identified as hydroxyl glutaric acid (**8**), malic acid (**13**), citraconic acid (methyl maleic acid) (**15**), dimethyl malate (**38**), and cinnamic acid (**21**) (Table 1).

#### 3.2.6. Fatty Acids and Derivatives

Eight fatty acids and one fatty acid ester were detected, in the case of compound (**116**), the fragmentation patterns were matched with 9,12,13- trihydroxy-octadecadienoic acid, the molecular ion at *m*/*z* 327.2178 [M − H]^−^ and the fragments at *m*/*z* 229 and *m*/*z* 171 pointed to the positions of hydroxyl groups of fatty acids (that is, at 12 and/or 13, 9 and/or 10th carbon) but it was not easy to assign the functional groups and double bonds depending on our data. Therefore, this compound was identified as trihydroxy-octadecadienoic acid. Similarly, trihydroxy-octadecanoic acid (**117**) has a molecular ion at *m*/*z* 329.2328 [M − H]^−^ and the base peak at *m*/*z* 211.1342, other fragments were detected at *m*/*z* 311, 229,171 which confirmed the skeleton of trihydroxy-octadecanoic acid. Other fatty acids were detected in the negative ionization mode as lichesterylic acid (methyl-oxo-heptadecanoic acid) (**134**) at *m*/*z* 297.243 [M − H]^−^, 10-hydroxyoctadeca-12,15-dienoic acid (**138**) at *m*/*z* 295.2282 [M − H]^−^, and hydroxyl docosanoate (**153**) at *m*/*z* 355.3217 [M − H]^−^. While, MS/MS fragmentation of compound (**150**) gave molecular ion at *m*/*z* 326.3796 [M − H]^+^ and characteristic fragment ions *m*/*z* 62.05 ([ethanolamine + H]^+^); *m*/*z* 308.2 ([M-H_2_O + H]^+^) and identified as N-oleoylethanolamine. In addition, two isomers of (17s)-hydroxy-docosa-pentaenoic acid were tentatively identified in all the examined *M. longipetala* plant parts (**149**; *m*/*z* 347.261 and **151**; *m*/*z* 347.256 [M + H]^+^). Moreover, one fatty acid ester was assigned (**152** at *m*/*z* 325.274 [M + H]^+^) as octadecenoic acid methyl ethyl ester.

#### 3.2.7. Lipids

Five phospholipids were detected in *M. longipetala* extracts; three glycerophosphoinositol lipids were identified mainly in the leaves (negative ion mode) and identified as octadecatrienoyl-glycerophosphoinositol (**125**, *m*/*z* 593.2724 [M − H]^−^), linoleoyl-glycerophosphoinositol (**126**, *m*/*z* 595.2885 [M − H]^−^), and palmitoyl-glycerophosphoinositol (**129**, *m*/*z* 571.2884 [M − H]^−^). In addition, two glycerophosphoglycerol lipids were identified in the root extract as hexadecenoyl-glycerophosphoglycerol at *m*/*z* 481.2568 [M − H]^−^ (**139**) and hexadecanoyl-glycerophosphoglycerol at *m*/*z* 483.2718 [M − H]^−^ (**146**).

Linoleoyl ethanolamide isomers (**133** and **145**; 322.2751 [M − H]^−^, 324.2901 [M + H]^+^) and palmitoyl ethanolamide (**148**; 300.2902 [M + H]^+^) are fatty amides that belong to the class of organic compounds known as *N*-acylethanolamines, in addition to dimethyl octadecenamide (**154**; 300.2902 [M + H]^+^). Lastly, one sulfoglycolipids was identified as hexadecanoyl glycerol-*O*-sulfo-rhamnoside at *m*/*z* 555.2844 [M − H]^−^ (**136**).

#### 3.2.8. Peptides

Eleven polypeptides were detected in the positive ionization mode (cluster D), thoroughly characterized for the root organ (Table 1, Figure 2). They were tentatively identified according to MS differences and fragmentation patterns, then further sequenced corresponding to [72].

### 3.3. Cytotoxicity

As expected, flower extract that showed the highest abundance of secondary metabolites revealed a significant cell viability inhibition of HCT-116 and HeLa cell lines growth, with LC_50_ values (24.8 ± 0.45 and 18.1 ± 0.42 µg/mL), compared to those of Doxorubicin (37.6 ± 0.21 and 26.1 ± 0.27 µg/mL), respectively. Similarly, the leaf extract inhibited the propagation of the HeLa cell line with an LC_50_ value of 29.6 ± 0.35 µg/mL. The three methanolic extracts did not show any cytotoxic effect on the HepG2 cell line (Supplementary Table S1). These findings summarize the relationships between the cytotoxic assessment of the three examined organs and the concentration of secondary metabolites, particularly flavonoids. Consequently, the present data indicates that the flower organ is responsible for activities reported before for aerial parts on the same species [8].

## 4. Conclusions

The current study provided a holistic overview of the constitutive metabolome of *M. longipetala,* an under-explored member of Brassicaceae. UPLC-HRMS/MS coupled to FBMN, and in silico fragmentation trees allowed for the annotation of 154 metabolites, belonging to phenolic acids, glucosinolates, flavonoids, lipids, peptides, and others. Furthermore, four previously unknown compounds were tentatively assigned as *O*-acetyl *O*-malonyl glucosides of quercetin (**103**), kaempferol (**108** and **112**), and isorhamnetin (**114**) based on their fragmentation pattern and their connectivity to their known analogs. Yet their full structure elucidation requires other spectroscopic techniques (i.e., NMR) after their isolation. Lastly, cytotoxicity assessment of the plant parts revealed that the flowers are effective against HeLa and HCT-116 cell lines suggesting that they are a potential resource of bioactive cytotoxic compounds. Future in vivo research should focus on the chemical modification and targeted delivery of these promising bioactive molecules to maximize their anticancer potential.

## Figures and Tables

**Table 1:** Metabolites identified in the aqueous methanol extracts of flowers, leaves, and roots from *Matthiola longipetala* subsp. *livida* via UPLC-HRMS-MS in negative and positive ionization modes.

No.	Chemical Class (Sub-Class)	RT	(M − H)^−^	(M + H)^+^	MS^2^		Root	Leaf	Flower	Tentatively Identification	Molecular Formula (Error in ppm)	Ref.
					Negative	Positive						
1	Amino acids	2.21	-	175.1193	-	158.0928, 128.0200, 116.0708, 86.9928 70.0651	+	+	++	Arginine	C_6_H_14_N_4_O_2_ (1.9)	[22]
2	Amino acids	2.41	-	116.0713	-	70.0660	+	+	++	Proline	C_5_H_9_NO_2_ (5.3)	[22]
3	Saccharides	2.45	341.1090	-	179.0565, 161.0469, 149.0471, 119.0348, 89.0245	-	+	+		Dihexoside	C_12_H_22_O_11_ (0.4)	[23]
4	Alkaloids and derivatives (alkaloids)	2.54	-	138.0505	-	122.0648, 110.0594, 96.0445	-	+	-	Trigonelline *	C_7_H_7_NO_2_ (−2.7)	[24]
5	Amino acids and derivatives	2.58	-	130.0861	-	86.0965, 84.0807, 70.0651	++	+	+	Methyl proline *	C_6_H_11_NO_2_ (1.0)	[22]
6	Amino acids and derivatives	2.59	-	144.1019	-	130.0502, 104.0294, 98.0602, 86.0965, 84.0807, 70.0651	+	++	+	Dimethyl proline	C_7_H_13_NO_2_ (0.3)	[22]
7	Glucosinolates (alkylglucosinolates)	2.60	434.0253	-	419.0020, 274.9899, 259.0128, 240.9670, 195.0335, 168.9510, 129.0253, 96.9602, 74.9912	-	+	+	++	Glucoraphenin	C_12_H_21_NO_10_S_3_ (0.4)	[25]
8	Organic acids (hydroxy acids)	2.70	147.0298	-	129.0193, 101.0244, 85.0295	-	t	++	++	Hydroxyglutaric acid	C_5_H_8_O_5_ (0.6)	[23]
9	Flavonoids (flavonol-O-glycoside)	2.72	-	873.2667	-	595.1675, 449.1086, 287.0556	-	+	+++	Km-*O*-rhamnosyl-arabinoside-*O*- rhamnosyl glucoside	C_38_H_48_O_23_ (0.2)	[8]
10	Glucosinolates (alkylglucosinolates)	2.83	-	176.0201	-	149.0595, 117.0335, 96.0443, 70.9950	-	+	+++	Raphenin *	C_6_H_9_NOS_2_ (−1.6)	[26]
11	Glucosinolates (alkylglucosinolates)	2.93	418.0299	-	259.0127, 224.9726, 176.0208, 96.9601, 74.9911	-	+++	+	-	Methylthiobutenyl- glucosinolate	C_12_H_21_NO_9_S_3_ (0.8)	[27]
12	Phenolic acids and derivatives (hydroxycinnamic acid glycosides)	3.02	487.1458	-	163.0422, 145.0295	-	-	-	+	Coumaric acid-*O*- dihexoside	C_21_H_28_O_13_ (0.4)	[28]
13	Organic acids (hydroxy acids)	3.60	133.0143	-	115.0039, 72.9935	-	t	+	+++	Malic acid	C_4_H_6_O_5_ (0.4)	[29]
14	Glucosinolates (alkylglucosinolates)	3.67	434.0254	-	419.0020, 274.9899, 259.0129, 240.9671, 195.0333, 168.9509, 129.0251, 96.9602, 74.9912	-	+	+	++	Glucoraphenin isomer	C_12_H_21_NO_10_S_3_ (0.2)	[30]
15	Organic acids (dicarboxylic acids)	3.75	-	131.0342	-	116.9329, 108.9868, 85.0282, 71.0153, 62.9826	+	+	+	Citraconic acid (methyl maleic acid)	C_5_H_6_O_4_ (−2.3)	[31]
16	Organic acids (carboxylic acids)	3.76	-	130.0868	-	86.0992, 84.0807, 85.0845, 70.0653	+	+	+	Pipecolic acid	C_6_H_11_NO_2_ (1.9)	[31]
17	Amino acids	3.79	-	132.1019	-	86.0965, 84.0809, 73.0648, 71.0128, 69.0699	+	+	+	Leucine/isoleucine	C_6_H_13_NO_2_ (0.1)	[22]
18	Glucosinolates (alkylglucosinolates)	3.92	418.0299	-	274.9898, 259.0127, 195.0335, 96.9602, 74.9912	-	+	-	-	Methylthiobutenyl-glucosinolate isomer	C_12_H_21_NO_9_S_3_ (0.2)	[27]
19	Phenolic acids and derivatives (hydroxybenzoic acid glucoside)	4.21	299.0770	-	137.0244, 93.0345	-	+	+	++	Hydroxy benzoic acid-*O*-hexoside	C_13_H_16_O_8_ (0.8)	[31]
20	Glucosinolates (alkylglucosinolates)	4.31	374.0582	-	259.0134, 195.0338, 96.9601, 74.9909	-	+	+	++	Butyl glucosinolate	C_11_H_21_NO_9_S_2_ (0.9)	[27]
21	Organic acids (cinnamic acids)	4.36	-	149.0600		105.0444, 104.0543, 79.0536	+	+	+	Cinnamic acid	C_9_H_8_O_2_ (−1.8)	[29]
22	Amino acids	4.37	164.0715	166.0630	147.0457, 103.0558, 72.0092	120.080, 103.0543, 93.0699	+	+	+	Phenylalanine *	C_9_H_11_NO_2_ (0.3)	[22]
23	Glucosinolates (alkylglucosinolates)	4.53	418.0299	-	274.9805, 259.0129, 224.9700, 195.0330, 176.0207, 96.9602, 74.9911	-	-	-	+	Methylthio-butenyl-glucosinolate isomer	C_12_H_21_NO_9_S_3_ (0.1)	[27]
24	Organic acids (carboxylic acids)	4.54	218.1036	220.1184	146.0823, 88.0406, 71.0501	202.1084, 184.0974, 172.1327, 158.0603, 142.0860, 124.0760, 98.0238, 72.0443	+	+	++	Pantothenic acid (vitamin B5)	C_9_H_17_NO_5_ (0.7)	[32]
25	Phenolic acids and derivatives (hydroxybenzoic acid glycosides)	4.70	315.0716	-	153.0186, 152.0113, 109.0297, 108.0225	-	+	+	+	Dihydroxybenzoic acid-*O*-hexoside	C_13_H_16_O_9_ (0.8)	[17]
26	Phenolic acids and derivatives (hydroxycinnamic acid glycosides)	4.81	517.1558	-	397.1158, 193.0508, 175.0401, 119.0345	-	t	-	+	Ferulic acid-*O*-dihexoside *	C_22_H_30_O_14_ (0.0)	[33]
27	Flavonoids (flavonol-O- glycosides)	5.01	-	773.2147	-	611.1624, 449.1084, 287.0555	t	+	+++	Km 3-*O*-sophroside- 7-*O*-glucoside	C_33_H_40_O_21_ (1.5)	[34]
28	Phenols (methoxyphenols)	5.02	-	151.0757	-	136.0605, 119.0496, 91.0543, 79.0550, 68.9826	t	+	+++	Methoxy- vinylphenol	C_9_H_10_O_2_ (−2.1)	[35]
29	Glucosinolates (alkylglucosinolates)	5.11	386.0582	-	274.9892, 259.0134, 195.0335, 96.9602, 74.9912	-	-	+	+++	Glucobrassicanapin	C_12_H_21_NO_9_S_2_ (0.5)	[28]
30	Iridoids (iridoids-O-glycosides)	5.13	375.1297	-	167.0709, 152.077	-	t	+	+++	Loganic acid	C_16_H_24_O_10_ (0.1)	[36]
31	Amino acids and derivatives	5.20	-	367.1504	-	349.1397, 332.1128, 303.1349, 276.1241, 258.1131, 229.0976, 202.064, 188.0713, 146.0603	+	+	+	Tryptophan *N*-hexoside	C_17_H_22_N_2_O_7_ (0.8)	[37]
32	Phenolic acids and derivatives (hydroxybenzoic acid glycosides)	5.30	329.0853	-	209.0445, 167.0350, 119.0342, 89.0245	-	t	+	+++	Vanillic acid- *O*-hexoside	C_14_H_18_O_9_ (0.2)	[17]
33	Flavonoids (flavonol-O- glycosides)	5.50	917.2648 ^a^	-	871.2510, 709.1991, 563.1405, 431.0940, 285.0369	-	-	-	+	Km -*O*-arabinosyl rhamnoside-*O*- rhamnoside-*O*- glucoside ^b^	C_38_H_48_O_23_ (0.5)	[8]
34	Amino acids	5.54	203.0824	205.0975	142.0664, 116.0503, 74.0248	188.0711, 170.0606, 159.0916, 146.0602, 132.0810, 118.0653	+	+	+	Tryptophan *	C_11_H_12_N_2_O_2_ (−0.2)	[22]
35	Glucosinolates (alkylglucosinolates)	5.65	418.0299	420.0457	274.9805, 259.0129, 224.9700, 195.0330, 176.0207, 96.9602, 74.9911	178.0360, 130.0324, 85.0282	+	+	++	Methylthio-butenyl-glucosinolate isomer	C_12_H_21_NO_9_S_3_ (0.6)	[27]
36	Phenolic acids and derivatives (hydroxybenzoic acid derivative)	5.81	246.9919	-	167.0350, 152.0116, 153.0452, 108.0219	-	+	+	+	Vanillic acid-sulfate	C_8_H_8_O_7_S (0.5)	
37	Phenolic acids and derivatives (hydroxybenzoic acid glycosides)	5.81	285.0614	-	153.019, 152.0112, 109.0292, 108.0220	-	+	+	+	Dihydroxybenzoic acid-*O*-pentoside	C_12_H_14_O_8_ (0.5)	[38]
38	Coumarins and derivatives (hydroxycoumarins)	5.91	-	163.0605	-	147.0446, 131.0497, 119.0494, 103.0544, 91.0541	t	+	+++	Hydroxycoumarin	C_9_H_6_O_3_ (−2.0)	[31]
39	Phenolic acids and derivatives (hydroxycinnamic acid glycosides)	5.94	487.1452	-	367.1031, 163.0397, 145.0293	-	t	+	+++	Coumaric acid- *O*-dihexoside	C_21_H_28_O_13_ (0.5)	[28]
40	Flavonoids (flavonol-*O*- glycosides)	6.10	609.1459	-	447.0927, 429.0825, 285.0404	-	t	++	++	Km 3-*O*- sophoroside *	C_27_H_30_O_16_ (0.3)	[39]
41	Phenolic acids and derivatives	6.11	395.0649	-	241.0023, 152.9863, 96.9602	-	+++	+	t	Dihydroxyphenylethanol-*O*-sulfoglucoside	C_14_H_20_O_11_S (0.9)	[40]
42	Flavonoids (flavonol-*O*- glycosides)	6.14	-	757.2199	-	449.1087, 287.0557	t	+	+++	Km-*O*-rutinoside- *O*-glucoside	C_33_H_40_O_20_ (1.1)	[41]
43	Phenolic acids and derivatives (hydroxycinnamic acid glycosides)	6.24	517.1556	-	397.115, 193.051, 175.039	-	t	+	+++	Ferulic acid-*O*- dihexoside isomer *	C_22_H_30_O_14_ (0.4)	[33]
44	Fatty acids	6.25	-	163.0601	-	131.0496, 119.0494, 103.0544, 91.0544, 77.0385	t	+	+++	Hydroxylhexanedioic acid * (Hydroxyadipic acid)	C_6_H_10_O_5_ (−2.7)	[42]
45	Coumarins and derivatives	6.31	-	207.065	-	193.0544, 179.0701, 147.0436, 119.0494, 91.0546	t	+	+++	Dimethoxycoumarin	C_11_H_10_O_4_ (2.1)	[43]
46	Phenolic acids and derivatives (hydroxycinnamic acid glycosides)	6.31	547.167	-	427.1245, 223.0618, 205.051, 190.0268, 179.0564	-	t	+	+++	Sinapic acid-*O*- dihexoside *	C_23_H_32_O_15_ (4.5)	[44]
47	Flavonoids (flavonol-*O*- glycosides)	6.39	871.2510	873.2667	709.1976, 563.1404, 447.0923, 431.0975, 285.105	711.1985, 595.1675, 449.1086, 433.1137, 287.0556	-	+	+++	Km 3-*O*-(2″-rhamnosyl)-arab-inoside-7-*O*-rhamnoside-4′-*O*-β-glucoside ^b^	C_38_H_48_O_23_ (0.7)	[8]
48	Flavonoids (Flavonol-*O*- glycosides)	6.39	593.1511	595.1665	285.0397, 284.0323	449.1088, 287.0559, 229.0868, 207.0658	t	+	+++	Km 3-*O*- rutinoside	C_27_H_30_O_15_ (0.4)	[31]
49	Flavonoids (flavonol-*O*- glycosides)	6.40	-	727.2088	-	449.1093, 287.0559	-	+	+++	Km *O*-arabinosyl rhamnoside-*O*- glucoside	C_32_H_38_O_19_ (1.1)	[31]
50	Flavonoids (flavonol-*O*- glycosides)	6.44	-	711.2146	-	433.1137, 287.0557	t	+	+++	Km-*O*-arabinosyl rhamnoside-*O*- rhamnoside	C_32_H_38_O_18_ (1.3)	[8]
51	Flavonoids (flavonol-*O*- glycosides)	6.54	741.1881	743.2038	609.1461, 579.1348, 463.0872, 447.0924, 301.0351	611.1608, 465.1035, 303.0504	t	+	+++	Qn-*O*-arabinosyl rhamnoside-*O*- glucoside	C_32_H_38_O_20_ (1.9)	[45]
52	Flavonoids (flavonol-*O*- glycosides)	6.74	739.2087	741.2247	593.1511, 447.0923, 431.0952, 285.0397, 284.0323	465.1035, 433.1137, 287.0556	t	+	+++	Km 3-*O*-rhamnoside 7-*O*-rutinoside *	C_33_H_40_O_19_ (0.5)	[46]
53	Flavonoids (flavonol-*O*- glycoside)	6.83	-	757.2199	-	479.1195, 463.1243, 317.0661	-	+	+++	Is *O*-arabinosyl rhamnoside-*O*- glucoside	C_33_H_40_O_20_ (1.8)	[31]
54	Phenolic derivatives (benzoyl derivatives)	6.88	-	137.0600	-	123.0394, 122.0365, 107.0500, 95.0415, 94.0415, 79.0544, 77.0387	t	+	+++	Methoxybenzaldehyde	C_8_H_8_O_2_ (2.5)	[47]
55	Flavonoids (flavonol-*O*- glycoside)	6.94	709.1973	-	563.1407, 431.0960, 285.0397, 284.0324	-	-	-	+	Km-*O*-rhamnosyl arabinoside-*O*- rhamnoside	C_32_H_38_O_18_ (0.3)	[8]
56	Coumarin and derivatives	6.98	-	207.0657	-	193.0544, 179.0710, 147.0447, 119.0494, 91.0543, 83.0495	t	+	+++	Dimethoxycoumarin isomer	C_11_H_10_O_4_ (2.0)	[43]
57	Phenolic acids and derivatives (hydroxycinnamic acid glycosides)	6.98	385.1141	-	223.0617, 205.0505, 190.0271, 179.0715, 164.0478	-	t	+	+++	Sinapic acid-*O*- hexoside *	C_17_H_22_O_10_ (0.1)	[17]
58	Flavonoids (flavonol-*O*- glycosides)	7.22	725.1937	727.2094	579.1357, 447.0898, 446.0850, 301.035	449.1086, 303.0507	t	t	+	Qn-*O*-rhamnosyl- arabinoside-*O*- rhamnoside	C_32_H_38_O_19_ (0.7)	[46]
59	Flavonoids (flavonol-*O*- glycosides)	7.29	609.1464	611.1615	463.0878, 447.0852, 301.0352, 285.0399	303.0505	t	+	+++	Qn 3-*O*-rhamnoside 7-*O*-glucoside	C_27_H_30_O_16_ (0.5)	[47,48]
60	Flavonoids (flavonol-*O*- glycosides)	7.41	609.1462	-	447.0927, 285.0404	-	t	+	+++	Km 3,7 di-*O*-glucoside *	C_27_H_30_O_16_ (0.3)	[39,49]
61	Phenolic derivatives (Benzoyl derivatives)	7.55	-	137.0601	-	123.0394, 122.0365, 107.0510, 95.0508, 94.0415	t	+	+++	Methoxybenzaldehyde isomer	C_8_H_8_O_2_ (−1.9)	[50]
62	Flavonoids (flavonol-*O*- glycosides)	7.65	579.1349	581.1510	447.0906, 446.0854, 433.0779, 301.0347	303.0506	t	t	+++	Qn 3-*O*-rhamnoside 7-*O*-arabinoside	C_26_H_28_O_15_ (1.3)	[51]
63	Flavonoids (flavonol-*O*- glycosides)	7.72	-	565.1552	-	287.0555	t	+	+++	Km 3-*O*-arabinoside-7-*O* rhamnoside	C_26_H_28_O_14_ (1.4)	[48]
64	Flavonoids (flavonol-*O*- glycosides)	7.73	709.1991	-	563.1407, 431.0960, 285.0397	-	-	-	+	Km *O*-rhamnosyl arabinoside-*O*- rhamnoside	C_32_H_38_O_18_	[8]
65	Flavonoids (flavonol-*O*- glycosides)	7.71	431.0960	433.1133	285.0397, 284.0324	287.0557	-	+	+++	Km 7-*O*- rhamnoside *	C_21_H_20_O_10_ (0.8)	[31]
66	Flavonoids (flavonol-*O*- glycosides)	7.82	709.1992	711.2139	563.1403, 431.0986, 285.0400	433.1138, 287.0556	t	+	+++	Km *O*-arabinosyl rhamnoside-*O*- rhamnoside ^b^	C_32_H_38_O_18_ (1.3)	[8]
67	Phenolic derivatives (Benzoyl derivatives)	7.86	357.1559	-	195.1032, 180.0784, 101.0236	-	t	+	+++	Trimethoxy benzaldehyde-*O*- hexoside	C_17_H_26_O_8_ (0.9)	[50]
68	Flavonoids (flavonol-*O*- glycosides)	7.91	739.2094	741.2243	285.0402	287.0557	t	+	+++	Km 3-*O*-(di-*O*- rhamnosyl) glucoside *	C_33_H_40_O_19_ (0.5)	[51]
69	Phenolic derivatives (benzaldehydes)	7.93	-	197.0813	-	137.0597, 105.0338, 79.0541	+	+	++	Trimethoxy benzaldehyde	C_10_H_12_O_4_ (−2.6)	[50]
70	Fatty acid derivatives (fatty acyl glycosides)	8.00		-	387.2039, 225.1504, 161.0458, 113.0258, 101.0247	-	t	++	++	Hydroxyjasmonic acid-*O*-hexoside (tuberonic acid- *O*-hexoside)	C_18_H_28_ O_9_ (2.0)	[52]
71	Flavonoids (flavonol-*O*- glycosides)	8.04	623.1633	625.1774	477.1031, 461.1071, 315.0510	317.0662	-	-	+	Is 3-*O*-rhamnoside 7-*O*-glucoside	C_28_H_32_O_16_ (1.5)	[46]
72	Flavonoids (flavonol-*O*- glycosides)	8.14	563.1398	-	431.0903, 417.08, 285.0403	-	-	t	+	Km 3-*O*-rhamnoside 7-*O*-arabinoside	C_26_H_28_O_14_ (0.5)	[48]
73	Flavonoids (flavonol-*O*- glycosides)	8.19	609.1446	-	447.0918, 285.0396, 284.0318	-	-	+	+++	Km 3-*O*- gentobiosde	C_27_H_30_O_16_ (0.8)	[39]
74	Flavonoids (flavonol-*O*- glycosides)	8.39	461.1069	463.1240	315.0509, 314.0429	317.0661	+	+	++	Is 7-*O-* rhamnoside	C_22_H_22_O_11_ (1.8)	[47]
75	Benzoic acid	8.41	153.0192	-	109.0296, 81.0350	-	t	+	+++	Dihydroxybenzoic acid	C_7_H_6_O_4_ (0.9)	[53]
76	Flavonoids (flavonol-*O*- glycosides)	8.43	593.1509	595.1663	461.1069, 447.0928, 315.0509	317.0661	t	++	++	Is 3-*O*-rhamnoside 7-*O*-arabinoside	C_27_H_30_O_15_ (0.5)	[28]
77	Flavonoids (flavonol-*O*- glycosides)	8.45	609.1468	611.1614	301.0346	303.0511, 287.0552, 229.0500, 129.0554, 85.0289	t	+	+++	Qn 3-*O*-rutinoside (rutin) *	C_27_H_30_O_16_ (0.3)	[46]
78	Benzoic acids and derivatives	8.61	223.0246	-	179.0354, 135.0461, 109.0297	-	t	+	+++	Carboxyvinyl benzoic acid	C_10_H_8_O_6_ (0.9)	[23]
79	Coumarins and derivatives	8.63	-	455.1164	-	411.1268, 369.1162, 207.0938, 179.0701, 147.0494, 79.0283, 69.0334	-	+	+++	Dimethoxycoumarin-*O*-malonyl glucoside	C_20_H_22_O_12_ (4.4)	[43]
80	Phenolic derivatives	8.92	355.1401	-	221.0442, 161.0453, 139.0222, 119.0345, 101.0245, 89.0247, 71.0140, 59.0141	-	-	-	+	Hydroxy phenyl pentanoic acid-*O*- glucoside	C_17_H_24_O_8_ (0.6)	[54]
81	Flavonoids (flavonol-*O*- glycosides)	9.02	463.0878	-	301.0277, 300.0276	-	t	+	+++	Qn 3-*O*- glucoside *	C_21_H_20_O_12_ (0.2)	[34]
82	Flavonoids (flavonol-*O*- glycosides)	9.08	593.1580	595.1665	285.0396, 284.0325	287.0552	t	+++	+++	Km-*O*- rutinoside *	C_27_H_30_O_15_ (0.6)	[55]
83	Flavonoids (flavonol-*O*- glycosides)	9.26	623.1622	625.1770	315.0510	317.0661	t	++	+++	Is 3-*O*- rutinoside *	C_28_H_32_O_16_ (0.7)	[51]
84	Phenolic derivatives (Cinnamaldehyde)	9.4	207.0665	-	192.0429, 179.0536	-	t	+	+++	Sinapaldehyde	C_11_H_12_O_4_ (0.7)	[17]
85	Flavonoids (flavonol-*O*- acylglycosides)	9.45	549.0881	551.1040	505.0988, 463.0882, 301.0331, 300.0275, 271.0242	303.0507	t	+	+++	Qn 3-*O*- malonylglucoside *	C_24_H_22_O_15_ (1.8)	[56]
86	Flavonoids (flavonol-*O*- acylglycosides)	9.54	505.0979	-	463.0886, 301.0332, 300.0272, 271.0247, 255.0306	-	t	+	+++	Qn 3-*O*-acetyl- glucoside *	C_23_H_22_O_13_ (1.7)	[57]
87	Flavonoids (flavonoid aglycones)	9.59	-	287.0505	-	257.0450, 229.0114, 149.0140, 97.0287	t	++	++	Km *^,b^	C_15_H_10_O_6_ (0.2)	[57]
88	Flavonoids (flavonol-*O*- glycosides)	9.60	447.0931	449.1083	327.0528, 285.0387, 284.0325, 255.0299, 227.0345	287.0555	t	++	++	Km 3-*O*-glucoside (astragalin) *	C_21_H_20_O_11_ (0.8)	[47]
89	Flavonoids (flavonol-*O*- glycosides)	9.71	433.0755	-	301.0332, 300.0276	-	t	+	+++	Qn 3-*O*- arabinoside	C_20_H_18_O_11_ (0.4)	[58]
90	Flavonoids (flavonol-*O*- glycosides)	9.83	477.1035	479.1195	315.0481, 314.0430, 299.0199, 285.0397, 271.0249, 243.0299	317.0664	t	-	+	Is 3-*O*- glucoside *	C_22_H_22_O_12_ (0.8)	[49,58]
91	Phenolics and derivatives	9.92	223.0251	-	179.0351, 153.0194, 135.0452, 109.0297	-	t	+	+++	Carboxyvinyl benzoic acid isomer	C_10_H_8_O_6_ (0.6)	[23]
92	Flavonoids (flavone-*O*- glycosides)	10.07	431.0981	433.1128	269.0447	271.060	+++	t	t	Apigenin 7-*O*- glucoside *	C_21_H_20_O_10_ (0.3)	[52]
93	Phenolic acids and derivatives (hydroxycinnamic acids)	10.11	179.0354	-	135.0461, 109.0297	-	t	+	+++	Caffeic acid *	C_9_H_8_O_4_ (0.4)	[52]
94	Flavonoids (flavonol-*O*- acylglycosides)	10.15	505.0979	507.1142	463.0886, 301.0332, 300.0275	303.0507	t	+	+++	Qn 3-*O*-acetyl glucoside isomer	C_23_H_22_O_13_ (1.7)	[56]
95	Flavonoids (flavonol-*O*- acylglycosides)	10.25	533.0936	535.1091	285.0385, 284.0323	287.0557	t	++	++	Km 3-*O*- malonylglucoside	C_24_H_22_O_14_ (1.6)	[59,60]
96	Flavonoids (flavonol-*O*- acylglycosides)	10.46	-	565.1198	-	317.0662	t	+	-	Is 3-*O*- malonylglucoside	C_25_H_24_O_15_ (1.8)	[61]
97	Flavonoids (flavonol-*O*- acylglycosides)	10.47	519.1143	-	477.0989, 315.0502, 314.0429	-	t	+	+	Is 3-*O*-acetyl glucoside	C_24_H_24_O_13_ (2.1)	[62]
98	Flavonoids (flavonol-*O*- acylglycosides)	10.51	505.0979	507.1142	463.0886, 301.0338, 300.0275	303.0505	t	+	+++	Qn 3-*O*-acetyl glucoside isomer	C_23_H_22_O_13_ (1.7)	[56]
99	Phenolic derivatives (phenylpropanoid glycosides)	10.58	341.1242	-	161.0428, 133.0660, 101.0254	-	t	+	+++	Coniferyl alcohol-*O*- glucoside (coniferin)	C_16_H_22_O_8_ (0.2)	[63]
100	Flavonoids (flavonol-*O*- glycosides)	10.65	447.0925	-	315.0477, 314.0430, 299.0209	-	t	+	+++	Is 3-*O*- arabinoside	C_21_H_20_O_11_ (2.2)	[46]
101	Phenolic derivatives (phenylpropanoid glycosides)	10.91	371.1350	-	209.0441, 163.0767, 148.0531	-	t	+	+++	Sinapoyl alcohol-*O*-glucoside (syringin)	C_17_H_24_O_9_ (−0.6)	[64]
102	Flavonoids (flavonol-*O*- glycosides)	10.99	431.0980	-	285.0400, 284.0323, 155.0303, 227.0347	-	-	+	+++	Km 3-*O*- rhamnoside (afzelin) *	C_21_H_19_O_10_ (0.9)	[65]
103	Flavonoids (flavonol-*O*- acylglycosides)	11.01	591.0995	593.1148	547.1082, 505.0982, 301.0333, 300.0277	303.0506	t	+	+++	Qn 3-*O*-X_1_ acetyl-X_2_ malonyl glucoside	C_26_H_24_O_16_ (0.3)	
104	Flavonoids (flavonol-*O*- acylglycosides)	11.11	519.1121	521.1297	477.1016, 315.0428, 314.0430, 299.0192	317.0664, 287.0547	t	++	++	Is 3-*O*-acetyl glucoside isomer	C_24_H_24_O_13_ (1.5)	[62]
105	Flavonoids (flavonol-*O*- acylglycosides)	11.23	489.1035	491.1192	285.0385, 284.0327	287.0555	t	++	++	Km 3-*O*-acetyl glucoside	C_23_H_22_O_12_ (0.7)	[56]
106	Saccharides	11.33	405.09	-	241.0022, 152.9869, 96.9604	-	+	+	+	Thioglucose-penta acetate	C_16_H_22_O_10_S (1.4)	
107	Flavonoids (flavonol-*O*- acylglycosides)	11.44		521.1296		317.0661, 127.0391	t	++	+++	Is 3-*O*-acetyl glucoside isomer	C_24_H_25_O_13_ (0.8)	[62]
108	Flavonoids (flavonol-*O*- acylglycosides)	12.01	575.1044	577.1191	531.1144, 489.1029, 285.0385, 284.0321	287.0555, 255.0441, 127.0391	t	++	+++	Km 3-*O*-X_1_ acetyl-X_2_ malonyl glucoside	C_26_H_24_O_15_ (0.5)	
109	Flavonoids (flavonol-*O*- acylglycosides)	12.02	531.1138	-	489.1031, 285.0385, 284.325, 255.0295	-	t	+	+++	Km 3-*O*-diacetyl glucoside	C_25_H_24_O_13_ (0.7)	[62]
110	Flavonoids (flavonol-*O*- acylglycosides)	12.23	561.1236	-	519.1152, 477.1016 315.0428, 314.0427, 299.0198	-	t	+	+++	Is 3-*O*-diacetyl glucoside	C_26_H_26_O_14_ (0.7)	[62]
111	Flavonoids (flavonol-*O*- glycosides)	12.31	461.1085	-	299.0554, 298.0485, 283.0250	-	+	++	+	Rh 3-*O*- glucoside ^b^	C_22_H_22_O_11_ (1.9)	[8]
112	Flavonoids (flavonol-*O*- acylglycosides)	12.68	-	577.1201	-	449.0908, 287.0555	t	+	+++	Km 3-*O*-X_1_ acetyl-X_2 -_malonyl glucoside isomer	C_26_H_24_O_15_ (−2.2)	
113	Flavonoids (flavonol-*O*- acylglycosides)	12.86	547.1095	549.1246	505.0963, 299.0239, 298.0280, 271.0250, 163.0770	301.0713, 231.0520, 159.0292, 127.0396	+	+++	+	Rh 3-*O*-malonoyl glucoside	C_25_H_24_O_14_ (−1.5)	[61]
114	Flavonoids (flavonol-*O*- acylglycosides)	12.88	-	607.1295	-	317.0662, 302.0427, 287.0546, 255.0512, 231.0500, 127.0392, 109.0287	t	+	+++	Is 3-*O*-X_1_ acetyl X_2_ malonyl glucoside	C_27_H_26_O_16_ (0.3)	
115	Flavonoids (flavonol-*O*- acylglycosides)	12.90	561.1223	-	519.1147, 477.0989, 315.0502, 314.0430, 299.0205	-	-	-	+	Is 3-*O*-diacetyl glucoside (isomer II)	C_26_H_26_O_14_ (0.5)	[62]
116	Fatty acid	13.70	327.2178	-	291.1951, 229.1452, 211.1384, 171.1026, 85.0297	-	++	+	+	Trihydroxy- octadecadienoic acid *	C_18_H_32_O_5_ (0.8)	[66]
117	Fatty acid	14.64	329.2328	-	311.2220, 229.1442, 211.1342, 171.1025	-	+++	+	+	Trihydroxy- octadecanoic acid*	C_18_H_36_O_5_ (0.4)	[67]
118	Flavonoids (flavonoid aglycones)	15.87	-	373.1285	358.1051, 343.0819, 329.1048, 315.0864, 229.0576	-	t	+++	+	Pentamethoxyflavone (tangeretin) *	C_20_H_20_O_7_ (0.9)	[68]
119	Flavonoids (flavonoid aglycones)	17.21	-	373.1287	359.1180, 343.0821, 329.1024, 312.1006, 297.0771, 283.0981, 229.0553	-	t	+++	+	Pentamethoxyflavone isomer (sinensetin) *	C_20_H_20_O_7_ (1.2)	[68]
120	Flavonoids (biflavone)	17.08	551.098	-	519.0691, 457.0573, 431.0760, 389.0667	-	t	+++	+	Amentoflavone methyl ether	C_31_H_20_O_10_ (0.0)	[69]
121	Flavonoids (biflavone)	18.05	551.099	-	519.0727, 457.0560, 431.0769, 389.0660	-	t	+++	+	Amentoflavone methyl ether isomer	C_31_H_20_O_10_ (0.1)	[69]
122	Flavonoids (flavonoid aglycones)	18.47	-	403.1393	373.0923, 359.1129, 343.0824, 329.1024, 313.0703	-	t	+++	+	Hexamethoxyflavone (Irigenin trimethyl ether) *	C_21_H_22_O_8_ (1.3)	[68]
123	Flavonoids (flavonoid aglycones)	19.29	-	433.1492	419.1299, 418.1265, 403.1029, 385.0914	-	t	+++	+	Heptamethoxyflavone (Nobiletin) *	C_22_H_24_O_9_ (0.8)	[68]
124	Flavonoids (flavonoid aglycones)	19.88	-	373.1286	358.1057, 343.0822, 325.0715, 312.0995, 271.0609, 211.0249, 183.0300	-	t	+++	+	Pentamethoxyflavone *	C_20_H_20_O_7_ (1.1)	[68]
125	Phospholipids (lysoglycerophos- phoinositol)	19.92	593.2724	595.2888	413.2085, 315.0483, 277.2171, 241.0119, 152.9966	335.2586, 261.2222, 243.2124, 184.0707, 155.0107, 81.0697	++	+	++	Octadecatrienoyl-glycero-phosphoinositol	C_27_H_47_O_12_P (0.4)	[70]
126	Phospholipids (lysoglycerophos- phoinositol)	21.71	595.2885	-	415.2244, 315.0475, 279.2329, 241.0116, 152.9960	-	++	+	++	Linoleoyl- glycero- phosphoinositol	C_27_H_49_O_12_P (0.2)	[70]
127	Diterpenes	21.82	-	283.1698	-	265.1586, 223.1485, 197.1330, 183.1205	+	+	++	Miltirone *	C_19_H_22_O_2_ (−1.0)	[71]
128	Fatty amides	22.41	-	298.346	-	281.0533, 245.1075, 227.0968, 74.0965	t	+	+++	*N*-hydroxyoleylamide *	C_18_H_35_NO_2_ (1.6)	
129	Phospholipids (lysoglycerophos- phoinositol)	22.88	571.2884	-	391.2254, 315.0487, 255.2329, 241.0119, 152.9959	-	+++	+	+	Palmitoyl- glycero-phosphoinositol *	C_25_H_49_O_12_P (−1.7)	[70]
130	Peptides	23.12	-	643.2734	-	586.2621, 583.2526, 529.2143, 523.2311, 381.2094, 311.1647, 293.1540, 265.1591, 247.1489, 205.1966, 182.1016, 147.0811, 133.1029, 89.0603	+	-	-	Glycyl-glycyl- phenylalanyl- alanyl- glutamyl-tyrosine	C_30_H_38_N_6_O_10_ (−1.8)	[72]
131	Peptides	23.94	-	657.2866	-	597.2677, 537.2455, 507.2379, 343.132, 311.1642, 247.1480, 205.1966, 181.1016 166.0754, 147.0811 133.1029	+	-	-	Serinyl- serinyl- glycyl- tyrosyl- phenylalanyl- proline	C_31_H_40_N_6_O_10_ (−1.4)	[72]
132	Peptides	24.00	-	691.2720	-	631.2520, 571.2318, 541.2204, 495.1999, 453.1890, 393.1681, 353.1750, 311.1641, 293.1539, 265.1591, 247.1485, 223.1123, 133.0858, 91.0540	+	-	-	Serinyl- phenylalanyl- glycyl- glutamyl- aspartyl- histidine	C_34_H_38_N_6_O_10_ (−1.5)	[72]
133	Fatty amides	24.43	-	322.2751	-	304.2645, 135.0326, 107.0862, 95.0860	+++	t	+	α-Linolenoyl ethanolamide *	C_20_H_35_NO_2_ (0.5)	[73]
134	Fatty acids	24.52	297.243		279.2334, 183.0120	-	+++	t	+	Methyl-oxo-heptadecanoic acid (lichesterylic acid) *	C_18_H_34_O_3_ (0.6)	
135	Peptides	24.61	-	685.29	-	625.2624, 565.2402, 353.1726, 293.1542, 247.1489, 181.1005, 182.0998, 147.0851, 119.0874, 106.0743	+	-	-	Tryptophyl-glutamyl-tyrosyl-serinyl-threonine	C_32_H_40_N_6_O_11_ (−1.8)	[72]
136	Lipid (sulfoglycolipids)	24.79	555.2844	-	299.0446, 255.2331, 206.9963, 80.9655	-	++	+++	+	Hexadecanoyl glycerol-*O*- sulfo-rhamnoside	C_25_H_48_O_11_S (0.2)	[74]
137	Peptides	24.99	-	627.3956	-	369.1955, 351.1849, 333.1749, 313.2087, 277.1588, 182.1230, 166.1178, 106.4462, 91.0543, 97.1052, 75.0725	+	-	-	Tyrosyl-glycyl-glycyl phenylalanyl- serinyl-proline	C_30_H_38_N_6_O_9_ (−1.6)	[72]
138	Fatty acids	25.05	295.2282	-	279.2331	-	+++	t	t	Hydroxyoctadecadienoic acid *	C_18_H_32_O_3_ (1.0)	
139	Phospholipids (lysophosphatidylglycerols)	25.16	481.2568	-	253.2174, 245.0430, 227.0324, 152.9959	-	t	+++	+	Hexadecenoyl glycero-phospho- sn-glycerol *	C_22_H_43_O_9_P (0.6)	[70]
140	Peptides	25.36	-	699.2995	-	639.2786, 579.2571, 519.2348, 495.1999, 453.1890, 393.1681, 353.1750, 311.1641 293.1539, 265.1591, 247.1485, 223.1123, 133.0858, 91.0540	+	-	-	Tyrosyl- threonyl-valinyl-methionyl-tryptophan	C_34_H_46_N_6_O_8_S (−1.6)	[72]
141	Peptides	25.72	-	675.2624	-	618.2624, 455.2040, 421.1996, 295.1699, 277.1592, 267.1749, 249.1642, 205.1903, 107.0875, 91.0543	+	-	-	Glycyl-Serinyl-tyrosyl-tryptophyl-tyrosine	C_34_H_38_N_6_O_9_ (−1.3)	[72]
142	Peptides	25.75	-	593.2754	-	397.2016, 355.1903, 295.1695, 277.1592, 267.1747, 249.1640, 207.1173, 165.0915	+	-	-	Acetyl-tryptophyl-methyl-alanyl-aspartyl-phenylalaninamide	C_30_H_36_N_6_O_7_ (−1.8)	[72]
143	Peptides	25.82	-	641.2942	-	581.2734, 521.2517, 461.2302, 313.1501, 295.1700, 277.1592, 249.1640, 173.0965, 106.0743, 91.0543	+	-	-	Serinyl-serinyl- glycyl-prolinyl- phenylalanyl-phenylalanine	C_31_H_40_N_6_O_9_ (−1.6)	[72]
144	Peptides	26.03	-	633.2679	-	576.2636, 523.2266, 437.1942, 377.1728, 267.1752, 253.1588, 239.1456, 107.0864, 91.0544	+	-	-	Nicotinoyl- alanyl- alanyl tyrosyl- glycyl- phenylalanine	C_32_H_36_N_6_O_8_ (−1.8)	[72]
145	Fatty amides	26.26	-	324.29	306.2793, 263.2363 245.2256, 147.1161, 109.1010, 95.0857, 81.0698, 62.0599	-	+++	t	+	Linoleoyl ethanolamide *	C_20_H_37_NO_2_ (0.2)	[73]
146	Phospholipids (lysophosphatidylglycerols)	26.60	483.2718		227.0324, 152.9955	-	+	+	+	Hexadecanoyl glycerophospho- glycerol	C_22_H_45_O_9_P (0.2)	[70]
147	Peptides	27.16	-	617.2724	-	557.2543, 497.298, 421.1988, 361.1785, 321.2406, 297.1849, 279.1747, 251.1804, 209.1325, 91.0538	+	-	-	Tryptophyl-glutamyl-prolyl-tryptophan	C_18_H_37_NO_2_ (0.5)	[72]
148	Fatty amides	27.70	-	300.2902	283.2642, 242.2482, 109.1012, 95.0857 85.1013, 71.0855, 67.0545, 62.0598	-	+++	t	+	Palmitoyl ethanolamide *	C_18_H_37_NO_2_ (0.5)	[75]
149	Fatty acids	28.07	-	347.2610	-	275.1620, 235.1318, 195.1004, 179.9946, 95.0865	t	t	+++	Hydroxy- docosa-pentaenoic acid *	C_22_H_34_O_3_ (0.5)	[76]
150	Fatty acids	28.34	-	326.3796	-	308.2959, 107.0847, 95.0857, 81.0889, 62.0599	+++	t	+	N-Oleoylethanolamine *	C_20_H_39_NO_2_ (0.2)	[77]
151	Fatty acids	28.68	-	347.2560	-	-	t	t	+++	Hydroxy- docosa-pentaenoic acid isomer *	C_22_H_34_O_3_ (0.6)	[76]
152	Fatty esters	30.21	-	325.274	-	265.2527, 247.2421, 135.1169, 121.1013, 109.1013, 95.0856, 81.0700, 67.0540	t	+	+++	Octadecenoic acid methyl ethyl ester	C_20_H_36_O_3_ (1.2)	[78]
153	Fatty acids	32.10	355.3217	-	337.3118, 309.3161, 297.1527	-	+++	t	t	Hydroxyl docosanoate	C_22_H_44_O_3_ (0.2)	[79]
154	Fatty amides	33.72	-	310.3111	293.2853, 275.2741, 97.1015, 83.0857, 69.0700	-	+	+	+	Dimethyl-octadecenamide	C_20_H_39_NO (−2.1)	[77]

^a^; [M + FA − H]^−^, ^b^; compound reported before from *M. longipetala* subsp. *livida*, *; tentatively identified compounds reported by GNPS libraries, +++; very strong, ++; strong, +, present, t; trace, -; absent, Km; kaempferol, Qn; quercetin, Is; isorhamnetin, Rh; rhamnocitrin.

## Data Availability

The designed MNs and parameters can be retrieved via the following links: https://gnps.ucsd.edu/ProteoSAFe/status.jsp?task=a88f28d3d0514197b8a14b54e60e9a13 accessed on 28 December 2019 for the positive network and https://gnps.ucsd.edu/ProteoSAFe/status.jsp?task=c8bb90ad81564de9b0d8032c016cc140 accessed on 28 December 2019 for the negative network.

## Supplementary Materials

The following supporting information can be downloaded at: https://www.mdpi.com/article/10.3390/metabo13080909/s1, Figure S1: The base peak chromatograms of *Matthiola longipetala* subsp. *livida* extracts: flowers (purple), leaves (green), and roots (yellow) in the negative ionization mode (A) and the positive ionization mode (B); Figure S2: Proposed fragmentation scheme and MS2 spectra (negative ionization mode) of A (isorhamnetin 3-*O*-glucoside, **90**), B (isorhamnetin 3-*O*-acetyl glucoside **96** and **104**), and C (isorhamnetin 3-*O*-diacetyl glucoside, **110** and **115**); Figure S3: Proposed fragmentation scheme and MS^2^ spectrum (negative ionization mode) of quercetin 3-*O*-X_1_ acetyl -X_2_ malonyl glucoside, **103**; Figure S4: Proposed fragmentation scheme and MS^2^ spectrum (negative ionization mode) of kaempferol 3-*O*-X_1_ acetyl -X_2_ malonyl glucoside, **108**; Figure S5: Proposed fragmentation scheme and MS^2^ spectrum (positive ionization mode) of isorhamnetin 3-*O*-X_1_ acetyl -X_2_ malonyl glucoside, **114**. Table S1: LC_50_ values (µg/mL) of the cell viability inhibition of *Matthiola longipetala* subsp. *livida* extracts on different cell lines.

## References

[1] Francis A., Lujan-Toro B.E., Warwick S.I., Macklin J.A., Martin S.L. (2021). Update on the Brassicaceae species checklist. Biodivers. Data J..

[2] Rahmani R., Bouajila J., Jouaidi M., Debouba M. (2020). African mustard (*Brassica tournefortii*) as source of nutrients and nutraceuticals properties. J. Food Sci..

[3] Elkhateeb A., El-Shabrawy M., Abdel-Rahman R.F., Marzouk M.M., El-Desoky A.H., Abdel-Hameed E.-S.S., Hussein S.R. (2019). LC-MS-based metabolomic profiling of *Lepidium coronopus* water extract, anti-inflammatory and analgesic activities, and chemosystematic significance. Med. Chem. Res..

[4] Bajkacz S., Ligor M., Baranowska I., Buszewski B. (2021). Separation and determination of chemopreventive phytochemicals of flavonoids from Brassicaceae plants. Molecules.

[5] Boulos L. (2009). Flora of Egypt Checklist.

[6] Hammami S., Ciavatta M., Ben Jannet H., Cimino G., Mighria Z. (2006). Three phenolic and a sterol glycoside identified for the first time in *Matthiola longipetala* growing in Tunisia. Croat. Chem. Acta.

[7] Tatsuzawa F. (2014). Acylated cyanidin 3-sambubioside-5-glucosides from the purple-violet flowers of *Matthiola longipetala* subsp. *bicornis* (Sm) PW Ball. (Brassicaceae). Phytochem. Lett..

[8] Marzouk M.M., Kawashty S.A., Ibrahim L.F., Saleh N.A., Al-Nowaihi A.-S.M. (2008). Two new kaempferol glycosides from *Matthiola longipetala* subsp. *livida* (Delile) Maire and carcinogenic evaluation of its extract. Nat. Prod. Commun..

[9] Abdelshafeek K.A., Abdelmohsen M.M., Hamed A., Shahat A.A. (2013). Investigation of some chemical constituents and antioxidant activity extracts of *Matthiola longipetala* subsp. *longipetala*. Chem. Nat. Compd..

[10] Akrout A., El Jani H., Zammouri T., Mighri H., Neffati M. (2010). Phytochemical screening and mineral contents of annual plants growing wild in the southern of Tunisia. J. Phytol..

[11] Baky M.H., Badawy M.T., Bakr A.F., Hegazi N.M., Abdellatif A., Farag M.A. (2021). Metabolome-based profiling of African baobab fruit (*Adansonia digitata* L.) using a multiplex approach of MS and NMR techniques in relation to its biological activity. RSC Adv..

[12] Wang M., Carver J.J., Phelan V.V., Sanchez L.M., Garg N., Peng Y., Nguyen D.D., Watrous J., Kapono C.A., Luzzatto-Knaan T. (2016). Sharing and community curation of mass spectrometry data with Global Natural Products Social Molecular Networking. Nat. Biotechnol..

[13] Hegazi N.M., Mohamed T.A., Saad H.H., Al-Hammady M.A., Hussien T.A., Hegazy M.E.F., Gross H. (2022). Molecular Network Guided Cataloging of the Secondary Metabolome of Selected Egyptian Red Sea Soft Corals. Mar. Drugs.

[14] Shabana M.M., Fathy F.I., Salama M.M., Hashem M. (2013). Cytotoxic and Antioxidant Activities of the Volatile Constituents of *Brassica tournefortii* Gouan: Growing in Egypt. Cancer Sci. Res..

[15] El-Amier Y.A., Zaghloul N.S., Abd-El Gawad A.M. (2023). Bioactive Chemical Constituents of *Matthiola longipetala* Extract Showed Antioxidant, Antibacterial, and Cytotoxic Potency. Separations.

[16] Hegazi N.M., Radwan R.A., Bakry S.M., Saad H.H. (2020). Molecular networking aided metabolomic profiling of beet leaves using three extraction solvents and in relation to its anti-obesity effects. J. Adv. Res..

[17] Hegazi N.M., Saad H.H., Marzouk M.M., Abdel Rahman M.F., El Bishbishy M.H., Zayed A., Ulber R., Ezzat S.M. (2021). Molecular networking leveraging the secondary metabolomes space of *Halophila stipulaceae* (Forsk.) Aschers. and *Thalassia hemprichii* (Ehrenb. ex Solms) Asch. in tandem with their chemosystematics and antidiabetic potentials. Mar. Drugs.

[18] Garg N., Kapono C.A., Lim Y.W., Koyama N., Vermeij M.J., Conrad D., Rohwer F., Dorrestein P.C. (2015). Mass spectral similarity for untargeted metabolomics data analysis of complex mixtures. Int. J. Mass Spectrom..

[19] Olmo-García L., Wendt K., Kessler N., Bajoub A., Fernández-Gutiérrez A., Baessmann C., Carrasco-Pancorbo A. (2019). Exploring the Capability of LC-MS and GC-MS Multi-Class Methods to Discriminate Virgin Olive Oils from Different Geographical Indications and to Identify Potential Origin Markers. Eur. J. Lipid Sci. Technol..

[20] Nothias L.-F., Petras D., Schmid R., Dührkop K., Rainer J., Sarvepalli A., Protsyuk I., Ernst M., Tsugawa H., Fleischauer M. (2020). Feature-based molecular networking in the GNPS analysis environment. Nat. Methods.

[21] Dührkop K., Shen H., Meusel M., Rousu J., Böcker S. (2015). Searching molecular structure databases with tandem mass spectra using CSI: FingerID. Proc. Natl. Acad. Sci. USA.

[22] Thiele B., Füllner K., Stein N., Oldiges M., Kuhn A.J., Hofmann D. (2008). Analysis of amino acids without derivatization in barley extracts by LC-MS-MS. Anal. Bioanal. Chem..

[23] Peng F., Liu Y., He C., Kong Y., Ouyang Q., Xie X., Liu T., Liu Z., Peng J. (2018). Prediction of platinum-based chemotherapy efficacy in lung cancer based on LC–MS metabolomics approach. J. Pharm. Biomed. Anal..

[24] Farid M.M., Yang X., Kuboyama T., Tohda C. (2020). Trigonelline recovers memory function in Alzheimer’s disease model mice: Evidence of brain penetration and target molecule. Sci. Rep..

[25] Hwang I.M., Park B., Dang Y.M., Kim S.-Y., Seo H.Y. (2019). Simultaneous direct determination of 15 glucosinolates in eight Brassica species by UHPLC-Q-Orbitrap-MS. Food Chem..

[26] Hanlon P.R., Barnes D.M. (2011). Phytochemical composition and biological activity of 8 varieties of radish (*Raphanus sativus* L.) sprouts and mature taproots. J. Food Sci..

[27] Wang H., Lin W., Shen G., Nomeir A.A., Khor T.-O., Kong A.-N. (2011). Development and validation of an LC-MS-MS method for the simultaneous determination of sulforaphane and its metabolites in rat plasma and its application in pharmacokinetic studies. J. Chromatogr. Sci..

[28] Marzouk M.M., Elkhateeb A., El-Shabrawy M., Farid M.M., Kawashty S.A., AbdelHameed E.-S.S., Hussein S.R. (2020). Chemical Profiling of *Farsetia aegyptia* Turra and *Farsetia longisiliqua* Decne. and their Chemosystematic Significance. Trop. J. Nat. Prod. Res..

[29] Farid M.M., Aboul Naser A.F., Salem M.M., Ahmed Y.R., Emam M., Hamed M.A. (2022). Chemical compositions of *Commiphora opobalsamum* stem bark to alleviate liver complications in streptozotocin-induced diabetes in rats: Role of oxidative stress and DNA damage. Biomarkers.

[30] Lee K.C., Chan W., Liang Z., Liu N., Zhao Z., Lee A.W.M., Cai Z. (2008). Rapid screening method for intact glucosinolates in Chinese medicinal herbs by using liquid chromatography coupled with electrospray ionization ion trap mass spectrometry in negative ion mode. Rapid Commun. Mass Spectrom..

[31] Farid M.M., Ibrahim F.M., Ragheb A.Y., Mohammed R.S., Hegazi N.M., Shabrawy M.O.E., Kawashty S.A., Marzouk M.M. (2022). Comprehensive phytochemical characterization of *Raphanus raphanistrum* L.: In vitro antioxidant and antihyperglycemic evaluation. Sci. Afri..

[32] Gill B.D., Saldo S.C., McGrail I.J., Wood J.E., Indyk H.E. (2020). Rapid Method for the Determination of Thiamine and Pantothenic Acid in Infant Formula and Milk-Based Nutritional Products by Liquid Chromatography—Tandem Mass Spectrometry. J. AOAC Int..

[33] Cartea M.E., Francisco M., Soengas P., Velasco P. (2010). Phenolic compounds in *Brassica* vegetables. Molecules.

[34] Hegazi N.M., Khattab A.R., Frolov A., Wessjohann L.A., Farag M.A. (2022). Authentication of saffron spice accessions from its common substitutes via a multiplex approach of UV/VIS fingerprints and UPLC/MS using molecular networking and chemometrics. Food Chem..

[35] Othman R., Ramya R., Hassan N.M., Kamoona S. (2020). GCTOF-MS and HPLC Identification of Phenolic Compounds with Different Fractional Extracts of *Lepironia articulata*. J. Pharm. Nutr. Sci..

[36] Aberham A., Pieri V., Croom Jr E.M., Ellmerer E., Stuppner H. (2011). Analysis of iridoids, secoiridoids and xanthones in *Centaurium erythraea*, *Frasera caroliniensis* and *Gentiana lutea* using LC–MS and RP-HPLC. J. Pharm. Biomed. Anal..

[37] Diem S., Bergmann J., Herderich M. (2000). Tryptophan-N-glucoside in fruits and fruit juices. J. Agric. Food Chem..

[38] Russo D., Kenny O., Smyth T.J., Milella L., Hossain M.B., Diop M.S., Rai D.K., Brunton N.P. (2013). Profiling of phytochemicals in tissues from *Sclerocarya birrea* by HPLC-MS and their link with antioxidant activity. Int. Sch. Res. Notices.

[39] Schmidt S., Zietz M., Schreiner M., Rohn S., Kroh L.W., Krumbein A. (2010). Identification of complex, naturally occurring flavonoid glycosides in kale (*Brassica oleracea* var. sabellica) by high-performance liquid chromatography diode-array detection/electrospray ionization multi-stage mass spectrometry. Rapid Commun. Mass Spectrom..

[40] Cao B., Zeng M., Hao F., Zhao C., Zhang B., Wu Y., Zhang Y., Li M., Feng W., Zheng X. (2023). Two polyphenols isolated from *Corallodiscus flabellata* BL Burtt ameliorate amyloid β-protein induced Alzheimer’s disease neuronal injury by improving mitochondrial homeostasis. Behav. Brain Res..

[41] Le Gall G., DuPont M.S., Mellon F.A., Davis A.L., Collins G.J., Verhoeyen M.E., Colquhoun I.J. (2003). Characterization and content of flavonoid glycosides in genetically modified tomato (*Lycopersicon esculentum*) fruits. J. Agric. Food Chem..

[42] Körver-Keularts I.M., Wang P., Waterval H.W., Kluijtmans L.A., Wevers R.A., Langhans C.D., Scott C., Habets D.D., Bierau J. (2018). Fast and accurate quantitative organic acid analysis with LC-QTOF/MS facilitates screening of patients for inborn errors of metabolism. J. Inherit Metab. Dis..

[43] Tine Y., Renucci F., Costa J., Wélé A., Paolini J. (2017). A method for LC-MS/MS profiling of coumarins in *Zanthoxylum zanthoxyloides* (Lam.) B. Zepernich and Timler extracts and essential oils. Molecules.

[44] Zhou L., Shi X., Ren X., Zhang J., Qin Z. (2016). Identification of phenolic components in the root and leaf of purple yam (*Dioscorea alata*) by UHPLC-DAD-ESI-MS/MS. Mod. Food Sci. Technol..

[45] Marzouk M.M., Ibrahim L.F., El-Hagrassi A.M., Fayed D.B., Elkhateeb A., Abdel-Hameed E.-S.S., Hussein S.R. (2020). Phenolic profiling and anti-Alzheimer’s evaluation of *Eremobium aegyptiacum*. Adv. Trad. Med..

[46] Marzouk M.M., Al-Nowaihi A.-S.M., Kawashty S.A., Saleh N.A. (2010). Chemosystematic studies on certain species of the family Brassicaceae (Cruciferae) in Egypt. Biochem. Syst. Ecol..

[47] Qin Y., Gao B., Shi H., Cao J., Yin C., Lu W., Yu L., Cheng Z. (2017). Characterization of flavonol mono-, di-, tri-and tetra-O-glycosides by ultra-performance liquid chromatography-electrospray ionization-quadrupole time-of-flight mass spectrometry and its application for identification of flavonol glycosides in *Viola tianschanica*. J. Pharm. Biomed. Anal..

[48] Marzouk M.M., Hussein S.R., Elkhateeb A., Farid M.M., Ibrahim L.F., Abdel-Hameed E.-S.S. (2016). Phenolic profiling of *Rorippa palustris* (L.) Besser (Brassicaceae) by LC-ESI-MS: Chemosystematic significance and cytotoxic activity. Asian Pac. J. Trop. Dis..

[49] Ablajan K., Abliz Z., Shang X.Y., He J.M., Zhang R.P., Shi J.G. (2006). Structural characterization of flavonol 3, 7-di-O-glycosides and determination of the glycosylation position by using negative ion electrospray ionization tandem mass spectrometry. J. Mass Spectrom..

[50] De Jager L.S., Perfetti G.A., Diachenko G.W. (2008). Comparison of headspace-SPME-GC–MS and LC–MS for the detection and quantification of coumarin, vanillin, and ethyl vanillin in vanilla extract products. Food Chem..

[51] Ragab N., El Sawi S., Marzouk M., El Halawany A., Sleem A., Farghaly A., Aboutabl E. (2021). Chemical characterization of *Melilotus messanensis* (L.) all.: Antioxidant, antidiabetic and antimutagenic effects in alloxan induced diabetic rats. Biocatal. Agric. Biotechnol..

[52] Marzouk M.M., Hussein S.R., Elkhateeb A., El-shabrawy M., Abdel-Hameed E.-S.S., Kawashty S.A. (2018). Comparative study of *Mentha* species growing wild in Egypt: LC-ESI-MS analysis and chemosystematic significance. J. Appl. Pharm. Sci..

[53] Bhagya N., Chandrashekar K. (2020). Identification and quantification of cytotoxic phenolic acids and flavonoids in *Ixora brachiata* by UHPLC-DAD and UHPLC-ESI-MS/MS. Int. J. Mass Spectrom..

[54] Ma C., Dunshea F.R., Suleria H.A. (2019). Lc-esi-qtof/ms characterization of phenolic compounds in palm fruits (jelly and fishtail palm) and their potential antioxidant activities. Antioxidants.

[55] Avula B., Bae J.Y., Wang Y.H., Wang M., Osman A.G., Smith K., Yuk J., Ali Z., Plumb R., Isaac G. (2020). Chemical profiling and characterization of phenolic acids, flavonoids, terpene glycosides from *Vangueria agrestis* using ultra-high-performance liquid chromatography/ion mobility quadrupole time-of-flight mass spectrometry and metabolomics approach. Biomed. Chromatogr..

[56] Oldoni T.L.C., Merlin N., Karling M., Carpes S.T., de Alencar S.M., Morales R.G.F., da Silva E.A., Pilau E.J. (2019). Bioguided extraction of phenolic compounds and UHPLC-ESI-Q-TOF-MS/MS characterization of extracts of *Moringa oleifera* leaves collected in Brazil. Food Res. Int..

[57] Lu Y.-H., Tian C.-R., Gao C.-Y., Wang X.-Y., Yang X., Chen Y.-X., Liu Z.-Z. (2019). Phenolic profile, antioxidant and enzyme inhibitory activities of *Ottelia acuminata*, an endemic plant from southwestern China. Ind. Crops Prod..

[58] Marzouk M.M., Elkhateeb A., Ibrahim L.F., Hussein S.R., Kawashty S.A. (2012). Two Cytotoxic Coumarin Glycosides from the aerial parts of *Diceratella elliptica* (DC.) Jonsell Growing in Egypt. Rec. Nat. Prod..

[59] Papetti A., Milanese C., Zanchi C., Gazzani G. (2014). HPLC–DAD–ESI/MSn characterization of environmentally friendly polyphenolic extract from *Raphanus sativus* L. var.“Cherry Belle” skin and stability of its red components. Food Res. Int..

[60] Cherfia R., Zaiter A., Akkal S., Chaimbault P., Abdelwahab A.B., Kirsch G., Chaouche N.K. (2020). New approach in the characterization of bioactive compounds isolated from *Calycotome spinosa* (L.) Link leaves by the use of negative electrospray ionization LITMSn, LC-ESI-MS/MS, as well as NMR analysis. Bioorg. Chem..

[61] Song S., Zheng X.P., Liu W.D., Du R.F., Feng Z.M., Zhang P.C., Bi L.F. (2013). Rapid identification of unstable acyl glucoside flavonoids of *Oxytropis racemosa* Turcz by high-performance liquid chromatography–diode array detection–electrospray ionisation/multi-stage mass spectrometry. Phytochem. Anal..

[62] Olennikov D., Kashchenko N. (2013). New isorhamnetin glycosides and other phenolic compounds from *Calendula officinalis*. Chem. Nat. Compd..

[63] Farid M.M., Ragheb A.Y., El-Shabrawy M., Marzouk M.M., Hussein S.R., Soliman A.A., Taha H., Kawashty S.A. (2020). GC-MS and LC-ESI-MS analysis of biologically active fractions from *Verbascum letourneuxii*; efficient protocol for in vitro propagation. Biocatal. Agric. Biotechnol..

[64] Plaza A., Montoro P., Benavides A., Pizza C., Piacente S. (2005). Phenylpropanoid glycosides from *Tynanthus panurensis*: Characterization and LC-MS quantitative analysis. J. Agric. Food Chem..

[65] Ibrahim L.F., Elkhateeb A., Marzouk M.M., Hussein S.R., Abdel-Hameed E.-S.S., Kassem M. (2016). Flavonoid investigation, LC–ESIMS profile and cytotoxic activity of *Raphanus raphanistrum* L. (Brassicaceae). J. Chem. Pharm. Res..

[66] Farag M.A., Otify A., Porzel A., Michel C.G., Elsayed A., Wessjohann L.A. (2016). Comparative metabolite profiling and fingerprinting of genus *Passiflora* leaves using a multiplex approach of UPLC-MS and NMR analyzed by chemometric tools. Anal. Bioanal. Chem..

[67] Tao Y., Cai H., Li W., Cai B. (2015). Ultrafiltration coupled with high-performance liquid chromatography and quadrupole-time-of-flight mass spectrometry for screening lipase binders from different extracts of *Dendrobium officinale*. Anal. Bioanal. Chem..

[68] Zhou D.-Y., Zhang X.-L., Xu Q., Xue X.-Y., Zhang F.-F., Liang X.-M. (2009). UPLC/Q-TOFMS/MS as a powerful technique for rapid identification of polymethoxylated flavones in *Fructus aurantii*. J. Pharm. Biomed. Anal..

[69] Swamy R.C., Kunert O., Schühly W., Bucar F., Ferreira D., Rani V.S., Kumar B.R., Appa Rao A.V.N. (2006). Structurally unique biflavonoids from *Selaginella chrysocaulos* and *Selaginella bryopteris*. Chem. Biodivers..

[70] Pulfer M., Murphy R.C. (2003). Electrospray mass spectrometry of phospholipids. Mass Spectrom. Rev..

[71] Guo L., Duan L., Dong X., Dou L.-L., Zhou P., Liu E.-H., Li P. (2014). A simple and sensitive LC–MS/MS method for determination of miltirone in rat plasma and its application to pharmacokinetic studies. J. Chromatogr. B.

[72] Perez-Miguez R., Plaza M., Castro-Puyana M., Marina M.L. (2019). Separation and identification of peptides in hydrolysed protein extracts from edible macroalgae by HPLC-ESI-QTOF/MS. Algal Res..

[73] Keereetaweep J., Blancaflor E.B., Hornung E., Feussner I., Chapman K.D. (2013). Ethanolamide oxylipins of linolenic acid can negatively regulate Arabidopsis seedling development. Plant Cell.

[74] Naumann I., Darsow K.H., Walter C., Lange H.A., Buchholz R. (2007). Identification of sulfoglycolipids from the alga *Porphyridium purpureum* by matrix-assisted laser desorption/ionisation quadrupole ion trap time-of-flight mass spectrometry. Rapid Commun. Mass Spectrom..

[75] Schreiber D., Harlfinger S., Nolden B.M., Gerth C.W., Jaehde U., Schömig E., Klosterkötter J., Giuffrida A., Astarita G., Piomelli D. (2007). Determination of anandamide and other fatty acyl ethanolamides in human serum by electrospray tandem mass spectrometry. Anal. Biochem..

[76] Meuronen T., Lankinen M.A., Fauland A., Shimizu B.-I., de Mello V.D., Laaksonen D.E., Wheelock C.E., Erkkilä A.T., Schwab U.S. (2020). Intake of *Camelina Sativa* oil and fatty fish alter the plasma lipid mediator profile in subjects with impaired glucose metabolism–a randomized controlled trial. Prostaglandins Leukot. Essent. Fatty Acids.

[77] Llorent-Martínez E.J., Spínola V., Gouveia S., Castilho P.C. (2015). HPLC-ESI-MSn characterization of phenolic compounds, terpenoid saponins, and other minor compounds in *Bituminaria bituminosa*. Ind. Crops Prod..

[78] Hussein S.R., Abdel Latif R.R., Marzouk M.M., Elkhateeb A., Mohammed R.S., Soliman A.A., Abdel-Hameed E.-S.S. (2018). Spectrometric analysis, phenolics isolation and cytotoxic activity of *Stipagrostis plumosa* (Family Poaceae). Chem. Pap..

[79] Wang F., Liigand J., Tian S., Arndt D., Greiner R., Wishart D.S. (2021). CFM-ID 4.0: More accurate ESI-MS/MS spectral prediction and compound identification. Anal. Chem..

[80] Mérillon J.M., Ramawat K.G. (2017). Glucosinolates.

[81] Andersen Q.M., Markham K.R. (2006). Flavonoids Chemistry, Biochemistry and Applications.

[82] Djoumbou Feunang Y., Eisner R., Knox C., Chepelev L., Hastings J., Owen G., Fahy E., Steinbeck C., Subramanian S., Bolton E. (2016). ClassyFire: Automated chemical classification with a comprehensive, computable taxonomy. J. Cheminformatics.

[83] Kawashty S.A., Hussein S.R., Marzouk M.M., Ibrahim L.F., Helal M.M.I., El Negomy S.I.M. (2012). Flavonoid constituents from *Morettia philaena* (Del.) DC and their antimicrobial activity. J. Appl. Sci. Res..

[84] Rogalewicz F., Hoppilliard Y., Ohanessian G. (2000). Fragmentation mechanisms of α-amino acids protonated under electrospray ionization: A collisional activation and ab initio theoretical study. Int. J. Mass Spectrom..

