# Supplementary material for: Discriminative Metabolomics Analysis and Cytotoxic Evaluation of Flowers, Leaves, and Roots Extracts of Matthiola longipetala subsp. livida
Source: Metabolites. 2023 Aug 3;13(8):909. doi: 10.3390/metabo13080909 (PMC10456503; doi:10.3390/metabo13080909)
Supplement: Supplementary file 1 [file metabolites-13-00909-s001.zip › metabolites-2502971-supplementary.pdf]

Supplementary Material

Discriminative Metabolomics Analysis and Cytotoxic Evaluation of Flowers, Leaves, and Roots Extracts of *Matthiola longipetala* subsp. *livida*

Mona M. Marzouk *, Nesrine M. Hegazi, Mona O. A. El Shabrawy, Mai M. Farid, Salwa A. Kawashty, Sameh R. Hussein and Nabiel A. M. Saleh

Phytochemistry and Plant Systematics Department, Division of Pharmaceutical Industries, National Research Centre, Cairo P.O. Box 12622, Egypt
* Correspondence: mm.marzouk@nrc.sci.eg; Tel.: +20-010000970022

List of content

Supplementary Figures **Page**

Figure S1: The base peak chromatograms of *Matthiola longipetala* subsp. *livida* extracts: flowers (purple), leaves (green), and roots (yellow) in the negative ionization mode (**A**) and the positive ionization mode (**B**) 2
Figure S2: Proposed fragmentation scheme and MS2 spectrum of **A** (isorhamnetin 3-*O*-glucoside, **90**), **B** (isorhamnetin 3-*O*-acetyl glucoside **96** & **104**), and **C** (isorhamnetin 3-*O*-diacetyl glucoside, **110** & **115**). *; The substitution position may vary 2
Figure S3: Proposed fragmentation scheme and MS^2 spectrum (negative mode) of quercetin 3-*O*-X_1 acetyl-X_2 malonyl glucoside, **103**. *; The substitution position may vary 3
Figure S4: Proposed fragmentation scheme and MS^2 spectrum (negative mode) of kaempferol 3-*O*-X_1 acetyl-X_2 malonyl glucoside, **108**. *; The substitution position may vary 3
Figure S5: Proposed fragmentation scheme and MS^2 spectrum (positive mode) of isorhamnetin 3-*O*-X_1 acetyl-X_2 malonyl glucoside, **114**. *; The substitution position may vary 3

Supplementary Table

Table S1: LC_{50} values (μg/mL) of the cell viability inhibition of *Matthiola longipetala* subsp. *livida* extracts on different cell lines 4

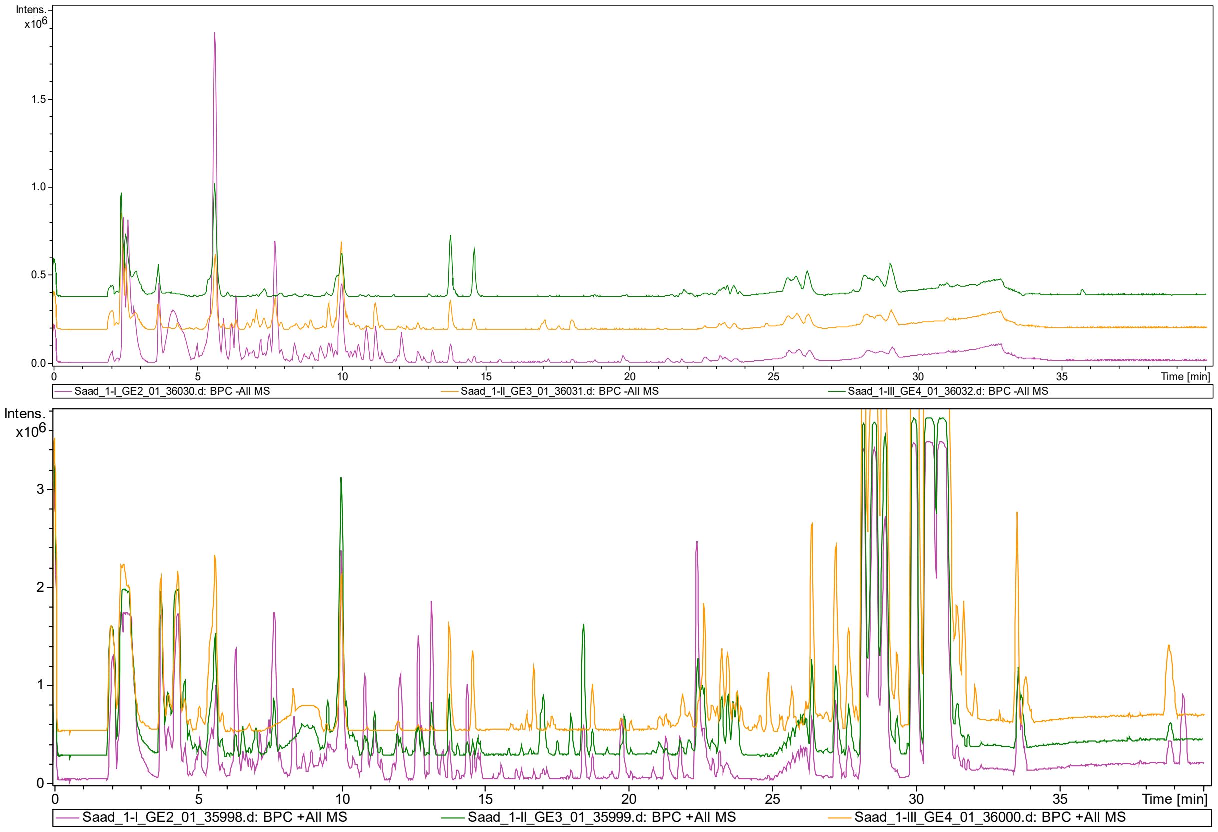

Figure S1. The base peak chromatograms of *Matthiola longipetala* subsp. *livida* extracts: flowers (purple), leaves (green), and roots (yellow) in the negative ionization mode (**A**) and the positive ionization mode (**B**)

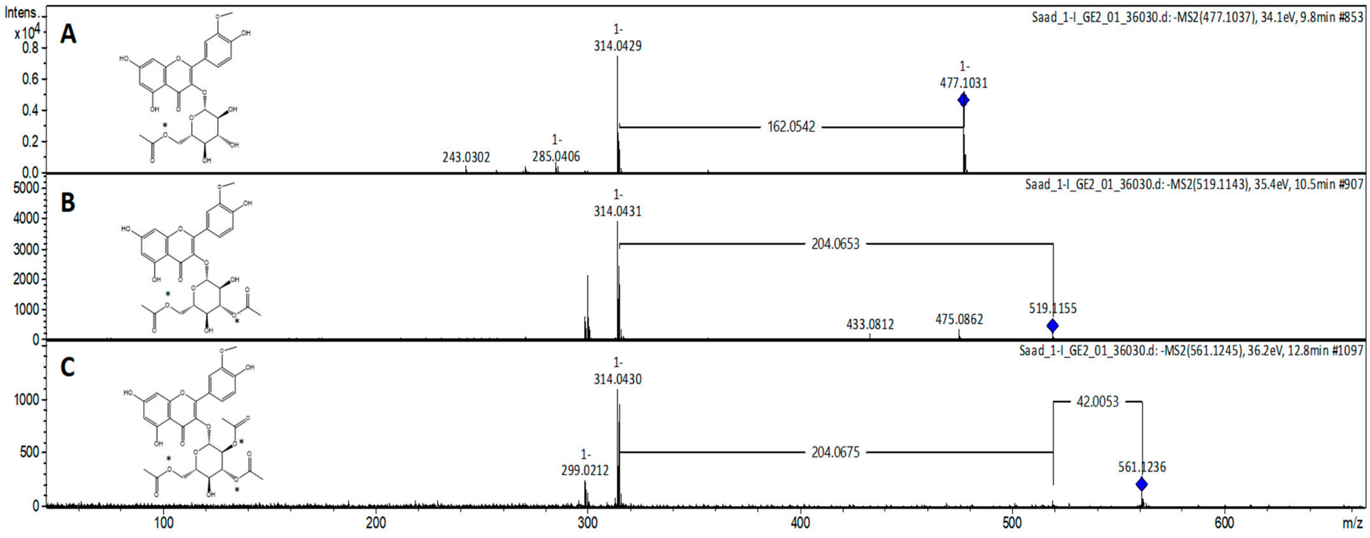

Figure S2. Proposed fragmentation scheme and MS2 spectrum of **A** (isorhamnetin 3-*O*-glucoside, **90**), **B** (isorhamnetin 3-*O*-acetyl glucoside **96** & **104**), and **C** (isorhamnetin 3-*O*-diacetyl glucoside, **110** & **115**). *; The substitution position may vary

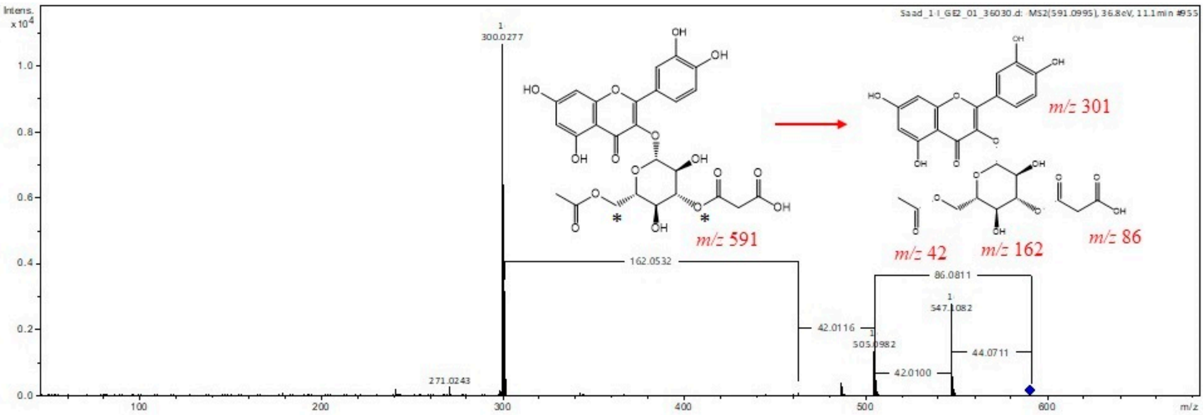

Figure S3. Proposed fragmentation scheme and MS² spectrum (negative mode) of quercetin 3-*O*-X$_1$ acetyl-X$_2$ malonyl glucoside, **103**. *; The substitution position may vary

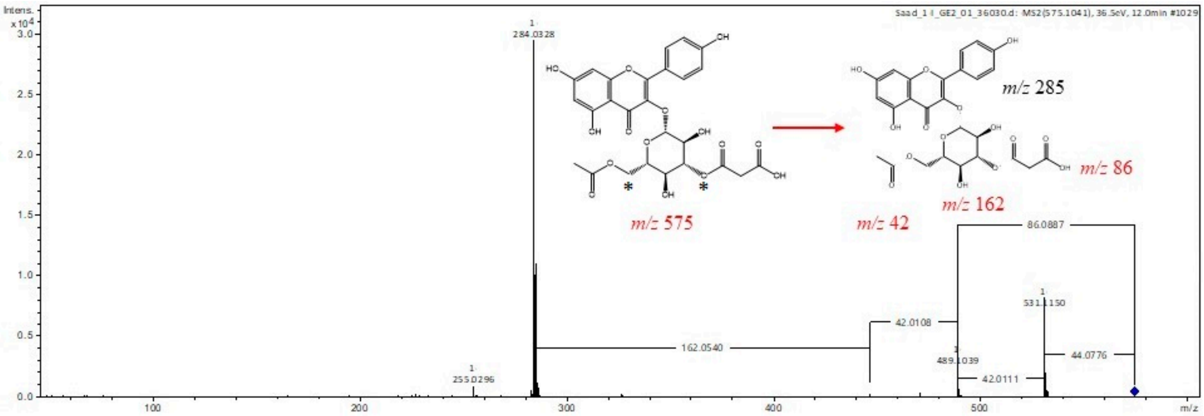

Figure S4. Proposed fragmentation scheme and MS² spectrum (negative mode) of kaempferol 3-*O*-X$_1$ acetyl-X$_2$ malonyl glucoside, **108**. *; The substitution position may vary.

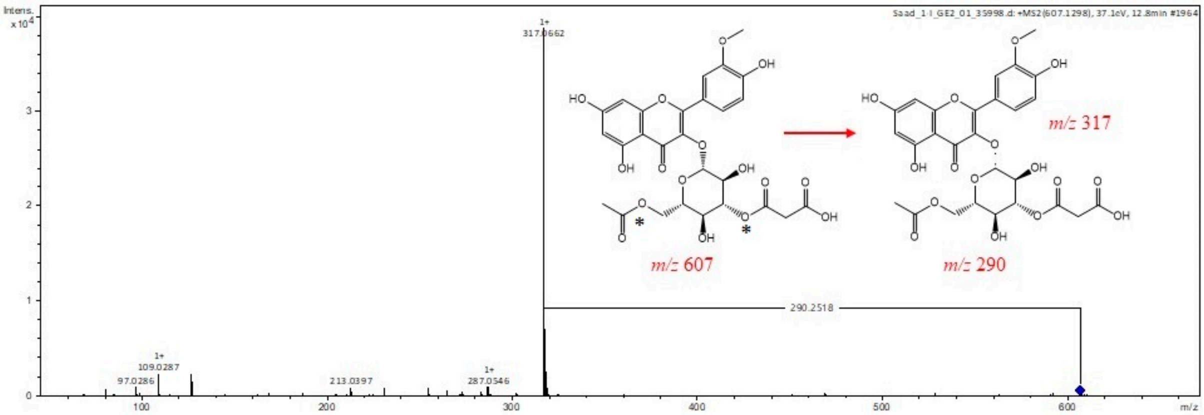

Figure S5. Proposed fragmentation scheme and MS² spectrum (positive mode) of isorhamnetin 3-*O*-X$_1$ acetyl-X$_2$ malonyl glucoside, **114**. *; The substitution position may vary.

Table S1. LC_{50} values (µg/mL) of the cell viability inhibition of *Matthiola longipetala* subsp. *livida* extracts on different cell lines

Plant organ extract	LC_{50} values (µg/mL)		
	**HeLa**	**HCT-116**	**HEPG2**
**Flowers**	18.1±0.42	24.8±0.45	-
**Leaves**	29.6±0.35	-	-
**Roots**	-	-	-
**Doxorubicin**	26.1±0.27	37.6±0.21	21.6±0.31
